# Dual PI3K- and mTOR-inhibitor PI-103 can either enhance or reduce the radiosensitizing effect of the Hsp90 inhibitor NVP-AUY922 in tumor cells: The role of drug-irradiation schedule

**DOI:** 10.18632/oncotarget.9501

**Published:** 2016-05-20

**Authors:** Cholpon S. Djuzenova, Vanessa Fiedler, Astrid Katzer, Konstanze Michel, Stefanie Deckert, Heiko Zimmermann, Vladimir L. Sukhorukov, Michael Flentje

**Affiliations:** ^1^ Department of Radiation Oncology, University Hospital of Würzburg, Würzburg, Germany; ^2^ Fraunhofer-Institut für Biomedizinische Technik, St. Ingbert and Lehrstuhl für Molekulare und Zelluläre Biotechnologie/Nanotechnologie, Universität des Saarlandes, Germany; ^3^ Department of Biotechnology and Biophysics, University of Würzburg, Würzburg, Germany; ^4^ Comprehensive Cancer Center Mainfranken, University of Würzburg, Würzburg, Germany

**Keywords:** cell cycle arrest, colony survival, DNA damage, histone γH2AX, radiation sensitivity

## Abstract

Inhibition of Hsp90 can increase the radiosensitivity of tumor cells. However, inhibition of Hsp90 alone induces the anti-apoptotic Hsp70 and thereby decreases radiosensitivity. Therefore, preventing Hsp70 induction can be a promising strategy for radiosensitization. PI-103, an inhibitor of PI3K and mTOR, has previously been shown to suppress the up-regulation of Hsp70. Here, we explore the impact of combining PI-103 with the Hsp90 inhibitor NVP-AUY922 in irradiated glioblastoma and colon carcinoma cells. We analyzed the cellular response to drug-irradiation treatments by colony-forming assay, expression of several marker proteins, cell cycle progression and induction/repair of DNA damage. Although PI-103, given 24 h prior to irradiation, slightly suppressed the NVP-AUY922-mediated up-regulation of Hsp70, it did not cause radiosensitization and even diminished the radiosensitizing effect of NVP-AUY922. This result can be explained by the activation of PI3K and ERK pathways along with G1-arrest at the time of irradiation. In sharp contrast, PI-103 not only exerted a radiosensitizing effect but also strongly enhanced the radiosensitization by NVP-AUY922 when both inhibitors were added 3 h before irradiation and kept in culture for 24 h. Possible reasons for the observed radiosensitization under this drug-irradiation schedule may be a down-regulation of PI3K and ERK pathways during or directly after irradiation, increased residual DNA damage and strong G2/M arrest 24 h thereafter. We conclude that duration of drug treatment before irradiation plays a key role in the concomitant targeting of PI3K/mTOR and Hsp90 in tumor cells.

## INTRODUCTION

Heat shock proteins 90 (Hsp90s) are ubiquitously and abundantly expressed polypeptides required for the energy-driven stabilization, conformation and function of a large number of cellular proteins, termed Hsp90 clients [1, 2]. Among many functions, Hsp90 clients contribute to the pathways involved in the induction of mitogen-activated protein kinases (MAPK) and nuclear factor-kappa B (NF-kB) [3, 4]. Hsp90 also stabilizes Raf-1, AKT, and ErbB2 proteins [5, 6], which are associated with protection against radiation-induced cell death [7, 8].

Considering the above mentioned functions of Hsp90, its inhibition can be a promising strategy for implementing a multi-target approach to radiosensitization. Indeed, a number of studies have already explored Hsp90 as a potential molecular target for tumor cells' radiosensitization [9–11]. Thus, various geldanamycin (17-DMAG or 17-AAG) or ansamycin derivatives (NVP-AUY922 or NVP-BEP800) significantly enhance the radiosensitivity of tumor cell lines derived from a variety of histologies, including glioma, prostate, and lung carcinoma [9–11].

At the same time, Hsp90 inhibition can induce up-regulation of Hsp90 itself along with its major co-chaperone Hsp70 [10, 12], which is known to promote cell survival by inhibiting both caspase-dependent and independent apoptotic pathways [13]. As a result, the anti-apoptotic action of heat shock proteins will reduce the radiosensitizing effect of Hsp90 inhibitors.

The up-regulation of Hsp90/Hsp70 levels can be temporarily suppressed by silencing gene expression via the introduction of synthetic siRNA into cells. However, neither pre-silencing of Hsp90 nor that of Hsp70 increases the radiosensitizing effect of the Hsp90 inhibitor NVP-AUY922 [14]. Alternatively, the induction of Hsp70 after pharmacological Hsp90 inhibition can be suppressed by concomitant PI3K inhibition with the pyridofuropyrimidine PI-103, a dual PI3K and mTOR inhibitor [15].

In the present study, we examined the ability of PI-103 to enhance the radiosensitizing effect of NVP-AUY922 in four tumor cell lines, including the glioblastoma GaMG and SNB19, and the colon carcinoma SW480 and SW48 cell lines. To this end, we analyzed control, drug-treated and irradiated cells by viability and colony-forming assays, induction and repair of radiation- and drug-induced DNA damage and cell-cycle distribution. We also assessed by Western blotting the expression levels of several marker proteins (Hsp90, Hsp70, PI3K, p-AKT, p-mTOR, p-4EBP1, p-S6, Raf-1, p-ERK1/2 etc.).

## RESULTS

### Cytotoxicity of PI-103 and NVP-AUY922

The cytotoxicity of PI-103 within the concentration range 0.01 – 20 μM in the presence of 200 nM NVP-AUY922 against *non-irradiated* tumor cells was studied by an ATP-based assay. The cellular ATP levels in cell samples treated with the drugs for 24 h were normalized against DMSO-treated controls and plotted *versus* PI-103 concentration (Supplementary Figure S1). With increasing PI-103 concentration, the mean ATP content in all cell lines decreased steadily depending on the cell line to 30–70% of the initial level after combined drug exposure. Based on these measurements, 2 μM of PI-103, causing 20–50% viability loss, was used for subsequent experiments. The selected PI-103 concentration is consistent with the previously reported data [15].

### Impact of PI-103 and NVP-AUY922 on Hsp90/Hsp70 expression and colony survival after irradiation

Next we compared two different drug-irradiation (IR) schedules for their radiosensitizing action on four tumor cell lines. In Schedule I, either PI-103 or NVP-AUY922, or both inhibitors were added to cell cultures for 24 h before IR (Supplementary Figure S2). In Schedule II, the inhibitors were added to cells 3 h before IR and kept in culture medium up to 24 h post-IR. The effects of drugs on Hsp90/Hsp70 expression and cell survival were analyzed by Western blotting and colony-forming assay, respectively.

**Figure 1:**
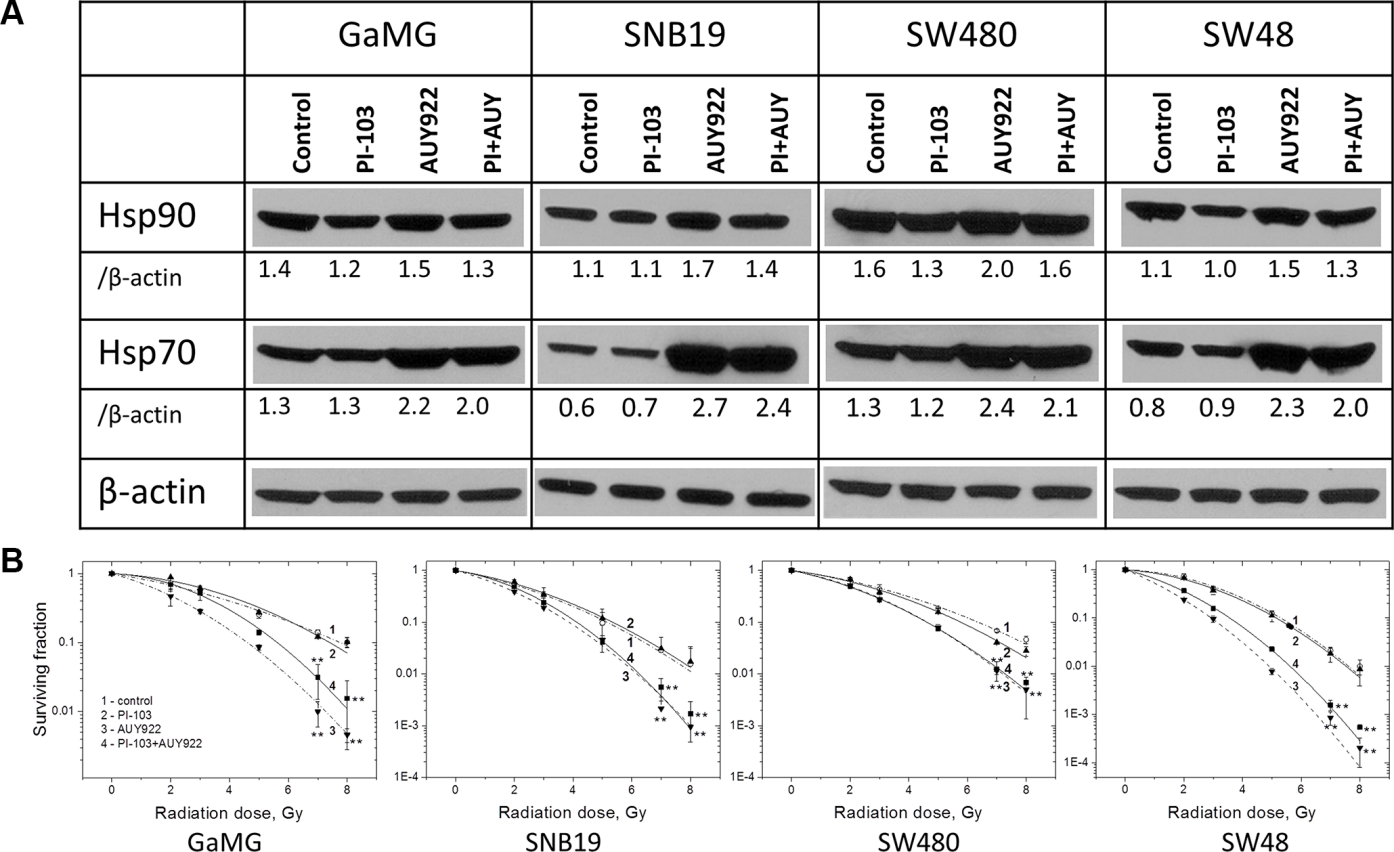
Long-term (24 h) pretreatment with both inhibitors moderately diminishes the up-regulation of Hsp70 without increasing the radiosensitizing ability of NVP-AUY922 (**A**) Western blot analysis of Hsp90 and Hsp70 protein levels in DMSO-treated controls and drug-treated (24 h) GaMG, SNB19, SW480 and SW48 cells. The protein/actin ratios are indicated by the numbers. (**B**) Clonogenic survival of irradiated tumor cell lines pre-treated for 24 h with PI-103, NVP-AUY922 or both. Cells were plated for the colony-forming test immediately after IR. Two weeks after, colonies containing at least 50 cells were scored as survivors. Data shown in part B derived from at least three independent experiments for each cell line in quadruplicate were pooled together and fitted by a LQ equation (Equation 1). The SD values are indicated by error bars. (Take note of the positions of curves 3 and 4). Student's *t*-test was conducted and considered significant at *p* < 0.05 (*), *p* < 0.01 (**), where the symbols * and # represent significant difference when compared either to vehicle or NVP-AUY922, respectively.

Figure 1A shows representative Western blots of Hsp90 and Hsp70 expressed in four tumor cell lines treated either with PI-103 or NVP-AUY922, or both substances for 24 h before IR according to Schedule I. As evident from the Figure, PI-103 alone exerted little (if any) effect on the expression levels of Hsp90 and Hsp70, as compared to untreated control. In contrast, treatment with the Hsp90 inhibitor NVP-AUY922 considerably increased the levels of Hsp70 (and to lesser extents of Hsp90) in all tested cell lines. For example, in NVP-AUY922-treated SNB19 cells, the expression of Hsp70 increased 4.5-fold, *i.e*. from the control level 0.6 to 2.7 a.u.

With the intention to prevent the up-regulation of Hsp70 induced by Hsp90 inhibition, we treated tumor cells simultaneously with NVP-AUY922 and PI-103 for 24 h according to Schedule I. As expected, concomitant treatment with two inhibitors suppressed to some extent the induction of Hsp90 and Hsp70 in all tested cell lines with respect to NVP-AUY922-treated samples (Figure 1A). However, the suppressive effect of PI-103 on the Hsp90/Hsp70 proteins was relatively weak in all tested cell lines. On average, Hsp90/Hsp70 expression in cells treated simultaneously with two substances was only by ~10–20% lower than in the corresponding samples treated with NVP-AUY922 alone.

We further analyzed whether the diminished up-regulation of Hsp90/Hsp70 in the presence of PI-103 and NVP-AUY922 affected the radiation sensitivity of tumor cells. Figure 1B shows the normalized survival responses of control and drug-treated cells plotted *versus* the radiation dose, along with the best fit curves of the LQ model (Equation 1) to the data. The plating efficiencies (PE) of non-irradiated cell samples, as well as the fitted parameters derived with the LQ model, including the surviving fraction at 2 Gy (SF2), the radiation dose required to reduce colony forming ability by 90% (D_10_) and the growth inhibition factor (I_10_) are summarized in Supplementary Table S1.

Contrary to the expectation, the combined treatment with PI-103 and NVP-AUY922 (Figure 1B, curves 4 for each cell line) according to Schedule I even slightly reduced the radiosensitizing effect of NVP-AUY922 (curves 3) in 2 (GaMG and SW48) out of 4 tested cell lines. Interestingly, PI-103 alone did not induce any radiosensitization in all tested cells lines, as evident from the closely overlapping curves 2 and 1 (control) in Figure 1B.

**Figure 2:**
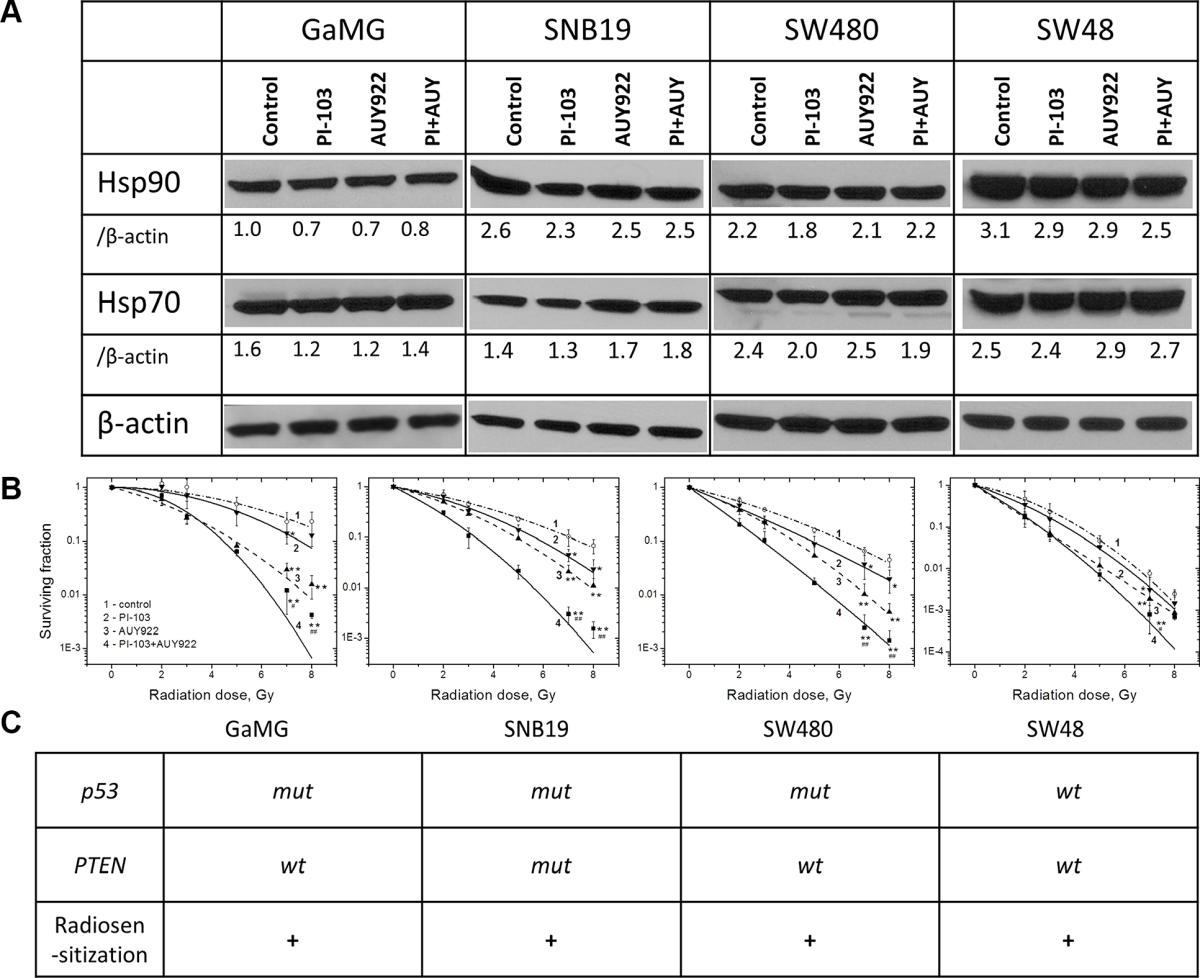
Short-term (3 h) treatment with both inhibitors does not upregulate the expression of Hsp90/Hsp70 but enhances the radiosensitizing effect of NVP-AUY922 in tumor cells (**A**) Western blot analysis of the expression levels of Hsp90 and Hsp70 proteins in DMSO-treated controls and drug-treated (3 h) GaMG, SNB19, SW480 and SW48 cells. (**B**) Clonogenic survival of irradiated tumor cell lines pre-treated for 3 h with PI-103, NVP-AUY922, or both. The cells were plated for the colony-forming test 24 h post-IR. (Take note of the positions of curves 3 and 4). (**C**) Mutational status of tested cell lines with respect to *p53* and *PTEN* as well as the radiosensitizing effect of PI-103. For further details, *see* Legend to Figure 1.

Since the reduced up-regulation of Hsp90/Hsp70 by PI-103 did not enhance the radiosensitizing ability of NVP-AUY922 under the conditions of Schedule I (Figure 1), we further attempted to completely avoid the drug-mediated up-regulation of Hsp90/Hsp70. To this end, we reduced the duration of drug treatment from 24 h (Schedule I) to 3 h before IR (Schedule II, Supplementary Figure S2). As seen from the Western blot detections in Figure 2A, the shorter exposure of cells to both inhibitors caused only little, if any, changes in the expression of Hsp90/Hsp70 proteins, with respect to control samples. At the same time, under this schedule, PI-103 moderately increased (Figure 2B, solid curves 4) the radiosensitizing effect of NVP-AUY922 (curves 3) in all tested cell lines. The enhancement of radiosensitization is also evident from the markedly reduced SF2 and D_10_ values (Supplementary Table S2) in irradiated cells treated with both drugs. Moreover, in contrast to a 24-h incubation, a 3-h treatment with PI-103 alone increased the radiation sensitivities of all tested cell lines (Figure 2B, curves 2), as compared to controls (curves 1). As seen in Figure 2C, the enhanced radiosensitization by a combination of drugs was independent of either *p53* or *PTEN* mutational status of the cell lines tested.

### Effects of PI-103 and NVP-AUY922 and/or radiation on multiple signaling pathways

**Figure 3:**
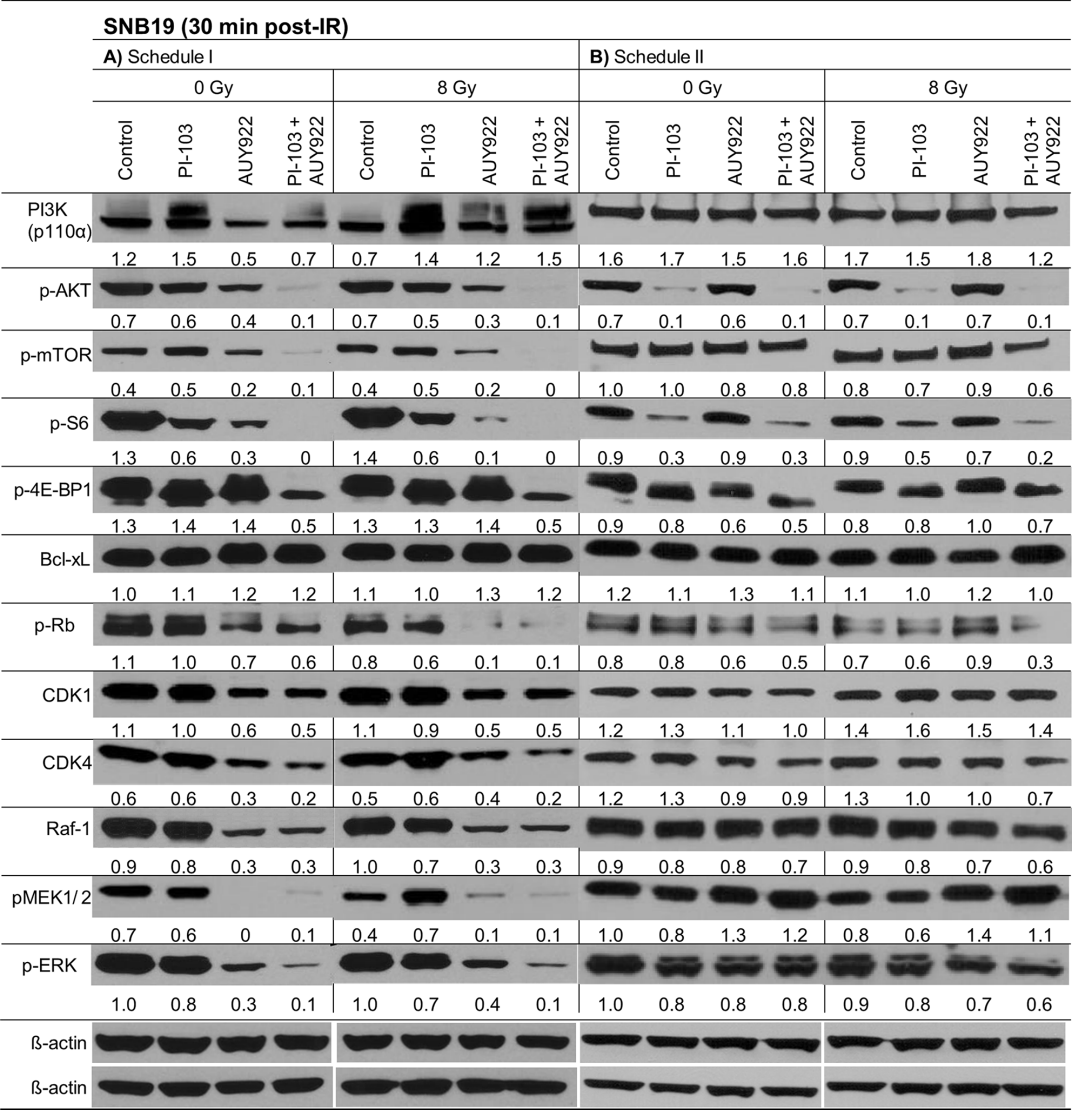
Representative Western blots of several marker proteins in SNB19 cells subjected to either 24-h (LHS) or 3-h (RHS) pretreatment with the inhibitors before IR Cell lysates were prepared 30 min after IR with 8 Gy. Each protein band was normalized to the intensity of β-actin used as loading control. The protein/β-actin ratios are denoted by the numbers. The experiment was repeated at least three times.

To elucidate the molecular basis for the observed schedule-dependent responses of drug-treated tumor cells to IR (Figures 1B, 2B), we analyzed the expression of several relevant marker proteins. Figure 3 shows exemplarily the Western blot data of control and drug-treated SNB19 cells probed for PI3K (p110α), pAKT, pmTOR, p-S6 and p4EBP1 and other indicated proteins 30 min after IR with 8 Gy. Samples shown on the left- and right-hand sides (LHS, RHS) of Figure 3 were treated according to Schedule I and II, respectively. The data for the other 3 tested cell lines at 30 min post-IR are shown in Supplementary Figures S3–S5.

As seen in Figure 3 (LHS column), a 24-h incubation with PI-103 substantially increased the expression of PI3K in SNB19 cells (*i.e*. from 1.2 to 1.5 a.u.), whereas upon a 3-h drug treatment using Schedule II (RHS of Figure 3) the PI3K level remained practically unchanged (1.6–1.7 a.u.). At the same time, the expression of p-AKT was strongly reduced from 0.7 a.u. in control to 0.1 a.u. in PI-103-treated sample after a 3-h drug treatment under Schedule II, with and without IR. In contrast, a 24-h treatment with PI-103 caused reactivation of the AKT function [16], *i.e*. p-AKT expression in both non-irradiated (0.6 a.u.) and irradiated (0.54 a.u.) samples almost recovered to the background level of ~0.7 a.u. (Figure 3, LHS). A 24-h treatment with the Hsp90 inhibitor alone reduced (~2 times) the expression of p-AKT (to 0.3–0.4 a.u.), whereas the combined treatment with both inhibitors for 24 h almost depleted p-AKT (~0.1 a.u.) independent of IR. After a short 3-h incubation with NVP-AUY922 alone, the expression levels of p-AKT and PI3K were almost unchanged as compared to the corresponding controls.

In addition to PI3K, we analyzed the expression of p-mTOR (a further target of PI-103) and its downstreams, ribosomal S6 and translational repressor 4EBP1 proteins, which are known to influence the cell-cycle progression and cell growth [17, 18]. The expression of p-mTOR was moderately increased after a 24-h incubation with PI-103 alone (Figure 3, LHS), but it was almost unchanged or even slightly reduced after a 3-h exposure to PI-103 in the cell sample treated with both substances for 3 h. Addition of the Hsp90 inhibitor alone or in combination with PI-103 under Schedule I (24-h treatment) strongly decreased or even depleted (~0–0.2 a.u.) the expression of p-mTOR. As a result, the pS6 protein was also strongly suppressed or depleted 30 minutes post-IR under both schedules (Figure 3 and Supplementary Figures S3–S5) in cells treated with PI-103 alone or in combination with NVP-AUY922. In contrast, expression of p-4E-BP1 was reduced to a much lesser extent (Figure 3 and Supplementary Figures S3–S5) and only after concomitant treatment with both substances, but also independent of the treatment schedule.

The lack of *PTEN* in *PTEN*-mutated cells, such as SNB19, usually leads to a compensatory activation of the PI3K pathway. Thus, activation of AKT in SNB19 cells typically results in an inhibition of Raf-1 and its downstreams MEK and ERK proteins through a cross-talk between the PI3K/AKT/mTOR and Ras/Raf/MEK/ERK (ERK signaling) pathways [19]. Besides this, as a result of Hsp90 inhibition, depletion of AKT can alter the ERK pathway. Normally the ERK pathway transmits signals from cell surface receptors to promote proliferation and survival programs, and it is frequently mutated in cancer cells [20, 21]. In fact, we found that a 24-h incubation with NVP-AUY922 alone or in combination with PI-103 reduced the expressions of Raf-1, p-ERK and pMEK (Figure 3 and Supplementary Figures S3–S5), which are known clients of Hsp90 protein, in all cell lines independent of IR. Interestingly, a 3-h incubation with PI-103 *increased* the expression of p-MEK1/2 after IR in most (except SW48) cell lines studied here. Another unexpected result was the up-regulation of Raf-1 in SW480 cells treated with PI-103 alone (Supplementary Figure S4). The most probable explanation for this result is that SW480 cells contain a mutation in *kRas*, which is directly implicated in the ERK signaling axis as well as in the PI3K pathway [22].

The next important finding was an increased expression of p-MEK1/2 in SNB19 (Figure 3) and GaMG (Supplementary Figure S3) cells which were pretreated for 3 h with the Hsp90 inhibitor alone, or in the presence of PI-103. The activation of MEK/ERK pathway by Hsp90 inhibition was also observed in several other studies [23, 24].

Analysis of the anti-apoptotic protein Bcl-xL revealed that a prolonged treatment (24 h) with the inhibitors (used either alone or in combination) caused a moderate up-regulation of Bcl-xL in SNB19 (Figure 3) as well as in GaMG and SW48 cell lines (Supplementary Figures S3, S5).

The Western blots of several cell cycle regulatory proteins, including cyclin-dependent kinases (Cdk1 and Cdk4) and pRb are illustrated for SNB19 cells in Figure 3. In the absence of inhibitors, irradiation of SNB19 cells (Figure 3) with 8 Gy resulted in a down-regulation of pRb and Cdk1 proteins after a 24-h pretreatment (Figure 3, LHS), without affecting their expression after a 3-h incubation period (Figure 3, RHS). It is also evident that a 24-h exposure to NVP-AUY922 alone and in combination with PI-103 caused a significant down-regulation of pRb, Cdk4 and Cdk1 proteins in non-irradiated SNB19 cells. In general, changes induced by combined drug-IR treatments were similar to those observed without IR, under both treatment schedules. Down-regulation of the G_1_/S regulatory protein Cdk4 was less pronounced in SNB19 (Figure 3) and GaMG (Supplementary Figure S3) cells, as compared to SW480 and SW48 cells (Supplementary Figures S4, S5). Cdk1 protein was decreased (GaMG, SNB19 and SW48) or even completely abolished (SW480) after Hsp90 inhibition alone or in combination with PI-103 after a 24-h pre-treatment (Schedule I). In contrast, a short incubation with the drugs (*i.e*. 3 h, Schedule II) did not significantly affect the expression of cell cycle-associated proteins, *i.e*. 30 min post-IR the expression of these proteins in irradiated cells was similar to that in non-irradiated samples.

**Figure 4:**
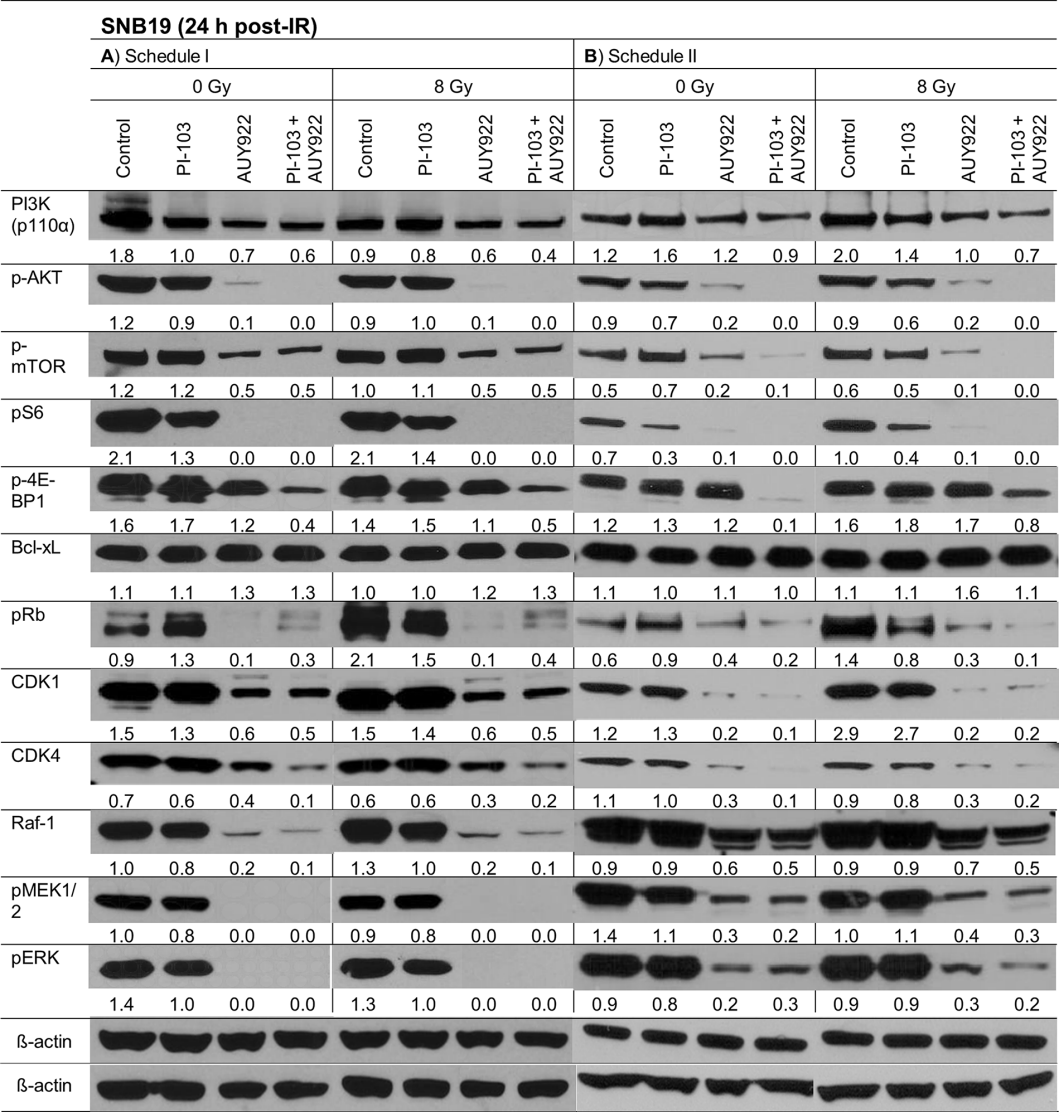
Representative Western blots of several marker proteins in SNB19 cells subjected to either 24-h (LHS) or 3-h (RHS) pretreatment with the drugs before IR Cell lysates were prepared 24 h after IR with 8 Gy. For details, *see* Legend to Figure 3.

In addition to the Western blot analysis performed 30 min post-IR, the above mentioned marker proteins were also detected at 24 h post-IR (Figure 4 and Supplementary Figures S6–S8). As seen in Figure 4, the striking difference between two schedules (24-h *versus* 3-h drug pre-treatment) in the expression of p-AKT and p-S6 in PI-103-treated cells observed at 30 min after IR (Figure 3) disappeared 24 h post-IR (Figure 4). The expression patterns of other tested proteins at 24 h after IR were similar under both schedules, *i.e*. they were either suppressed (p-4E-BP1, Cdk1, Cdk4) or completely depleted (p-AKT, p-S6, Raf-1, p-ERK, etc.) after treatment with NVP-AUY922 alone or in combination with PI-103. PI-103 alone strongly decreased the levels of p-S6 and p-4E-BP1 proteins. At the same time, PI-103 only moderately decreased the expression of the main proteins of the ERK-pathway, including Raf-1 and p-ERK.

The effects of PI-103 and NVP-AUY922 on the expression of most marker proteins in GaMG, SW480 and SW48 cell lines (Supplementary Figures S6–S8) at 24 h post-IR were qualitatively similar (except for the cell-cycle relevant proteins) to those observed in SNB19 cells under both schedules (Figure 4).

In addition, we detected the expression of non-phosphorylated forms of AKT, mTOR, and other proteins. As seen in Supplementary Figures S9–S16, contrary to the phosphorylated forms, the expression of non-phosphorylated forms of AKT, mTOR, 4E-BP1 and S6 was only moderately repressed after addition of the inhibitors. At the same time, the expression of MEK and ERK proteins was not changed at all.

### Assessment of late-stage apoptosis

**Figure 5:**
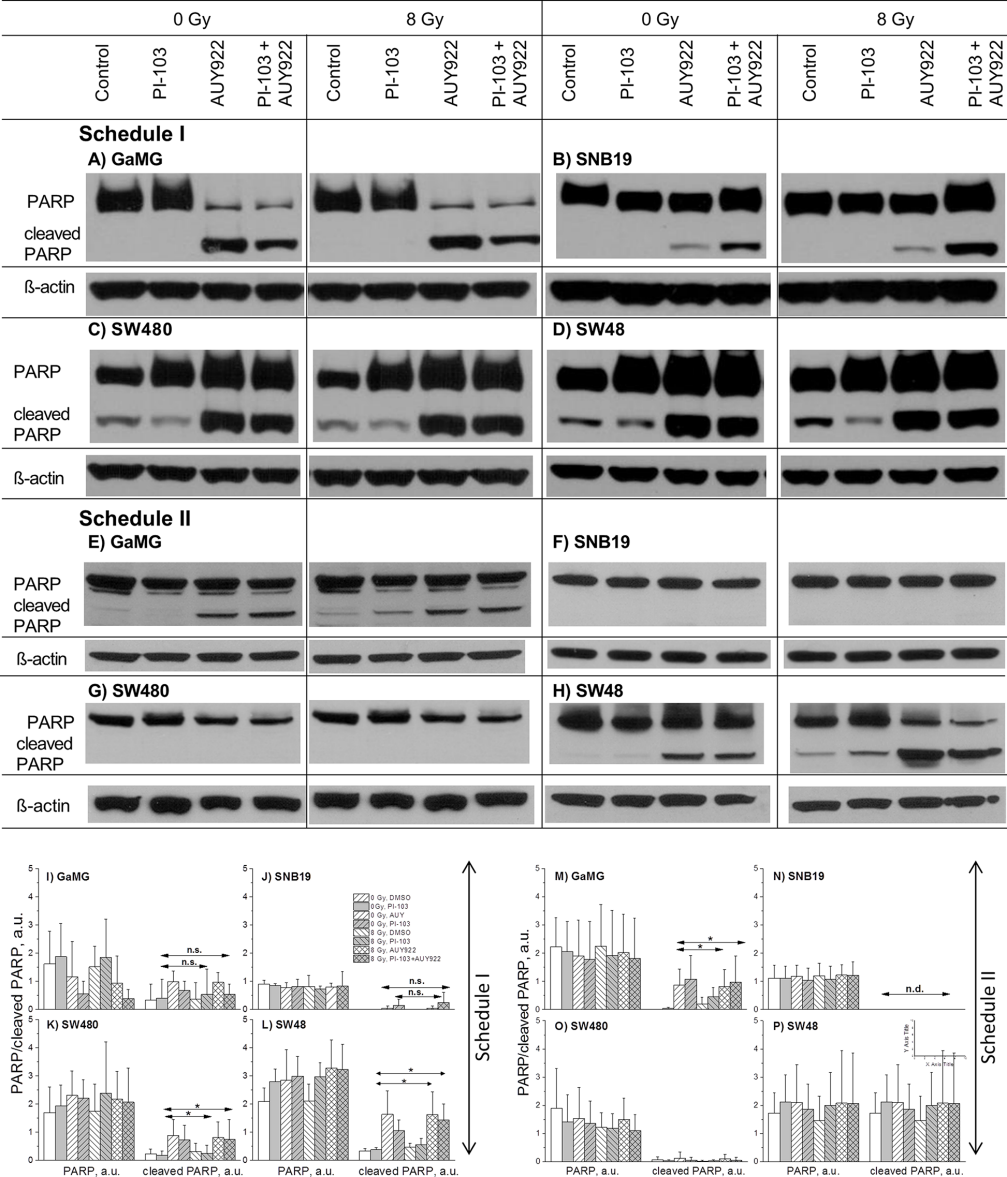
Representative Western blots (A-H) of PARP and cleaved PARP proteins in 4 tumor cell lines subjected to 24-h (Schedule I) or 3-h (Schedule II) pre-treatment with the drugs before IR The bar graphs are the means (± SE) of at least 3 independent experiments such as shown in parts I-P. Cellular lysates were prepared 24 h after IR with 8 Gy. For details, *see* Legend to Figure 3.

In efforts to identify the mechanisms underlying the increased radiation sensitivity of tumor cells after combined PI3K/mTOR/Hsp90-inhibition shown in Figure 2B, we also examined the expression of cleaved PARP, an established pro-apoptotic marker. Figure 5A–5H show exemplarily the expression of uncleaved and cleaved PARP in non-irradiated or irradiated cell samples detected 24 h after IR, either untreated or treated with inhibitors under both schedules. Increased levels of cleaved PARP were found under Schedule I in all cell lines treated with NVP-AUY922 alone or in a combination with PI-103 independent of IR. The extents of cleaved PARP induction, however, varied markedly among the 4 tested cell lines. The highest levels of cleaved PARP were observed in SW480 and SW48 cell lines (Figure 5C and 5D, respectively). Representative Western blots from at least 3 experiments are summarized in parts I-P of Figure 5. As seen in the bottom part of Figure 5, under Schedule II only 2 (GaMG and SW48) out of 4 tested cell lines showed apoptosis.

### Induction and decay of histone γH2AX

**Figure 6:**
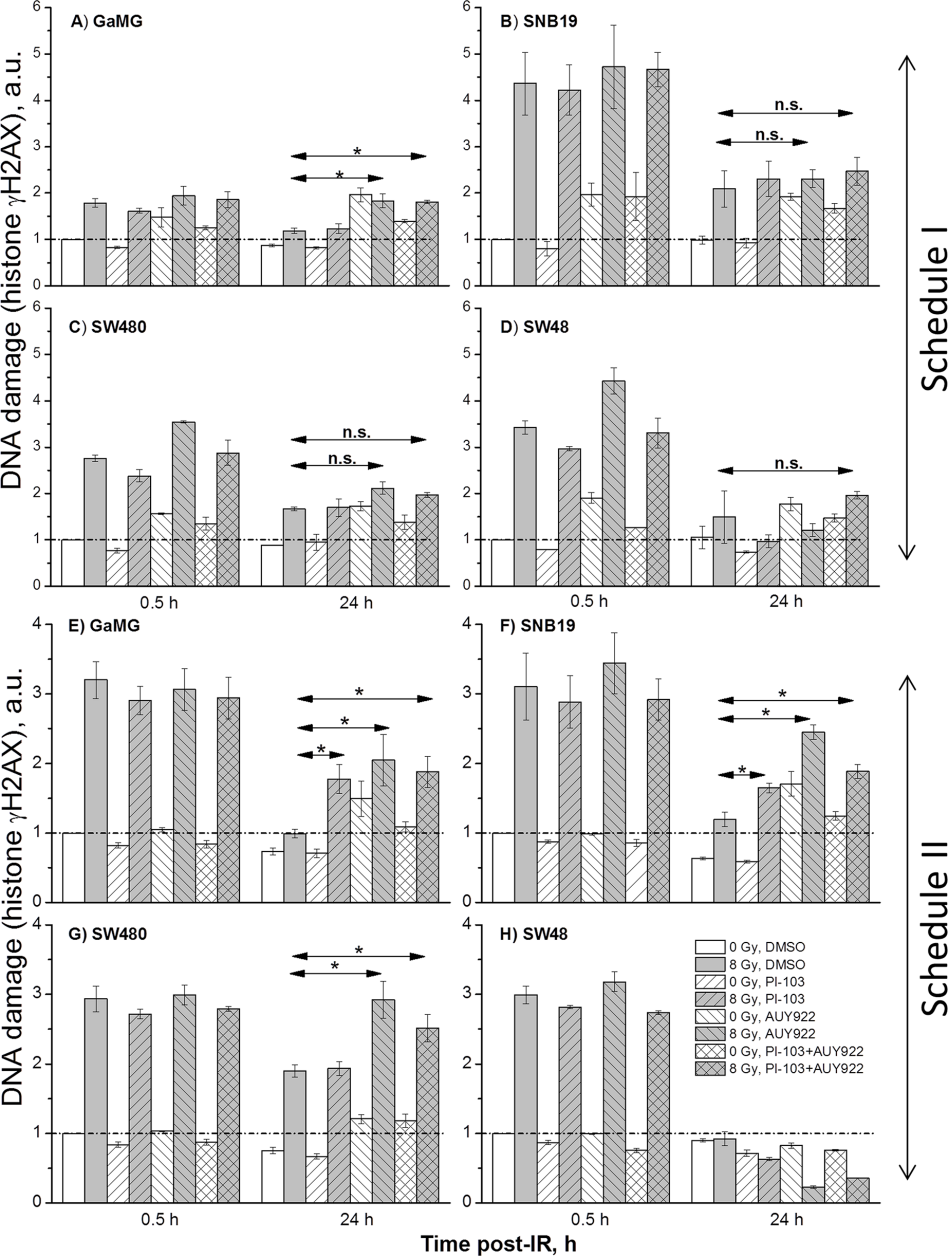
DNA damage in GaMG (A, E), SNB19 (B, F), SW480 (C, G) and SW48 (D, H) cells assessed by histone γH2AX and quantified by flow cytometry Top and bottom halves of the graph refers to the Schedule I and II, respectively. The bar graphs are the means (± SD) of at least 3 independent experiments such as shown in Supplementary Figure S17. The data of each cell line are normalized to the initial γH2AX content (at 0.5 h post-IR) detected in drug-free non-irradiated controls. “a.u.” means arbitrary units.

The induction of DNA double-strand breaks (DSBs), detected by the expression of phosphorylated histone H2AX [25], was measured 30 min and 24 h (Figure 6) after irradiation of tumor cells, non-treated or pre-treated with inhibitors (Figure 6 and Supplementary Figures S17–S19).

As evident from the representative flow cytograms (Supplementary Figure S17), the degree of the radiation-induced DNA damage in the colon carcinoma SW480 cells treated with PI-103 was even lower than in DMSO-treated controls under Schedule I (Figure S17A). Thus, 30 min after IR, PI-103-treated and irradiated SW480 cells exhibited even less DNA damage (2005 a.u. of γH2AX, Figure S17A) than did the corresponding irradiated drug-free controls (2408 a.u.). The highest DNA damage (3144 a.u.) was induced by NVP-AUY922 and IR, whereas a combination of substances caused less DNA damage (2327 a.u.) that was similar to that of the drug-free sample. Both substances given simultaneously under Schedule I did not noticeably affect the DNA damage repair process in SW480 tumor cells (Figure S17B), as suggested by the rapid reduction of γH2AX observed 24 h after IR (1709 a.u.), which was comparable to that in control (1523 a.u.).

On the other hand, if a combination of both substances was added according to Schedule II, the unrepaired DNA damage (Figures S17C, S17D) was much higher (~3100 a.u.) than in drug-free (~2500 a.u.) cells (Figure S17D). Moreover, although under Schedule II the initial levels of DNA damage 30 min after IR were similar in all samples (3800 ± 200 a.u., Figure S17C), the cells treated with NVP-AUY922 alone or in combination with PI-103 exhibited much higher residual γH2AX levels (~3500 and ~3100 a.u., respectively) 24 h after IR, as compared to the DNA damage levels in drug-free controls (~2500 a.u., Figure S17D). Latter findings suggest a negative impact of the inhibitors given under Schedule II on the DNA repair mechanisms.

As seen in Supplementary Figure S17, NVP-AUY922-treated tumor cell samples exhibited two distinct subpopulations (responding and non-responding to the drug) differing markedly in their γH2AX expression, as well as in the amounts of cells in each subpopulation. Given that all cell lines used here had similar cell-cycle distributions before drug treatment, the γH2AX expression mediated by the drugs alone (without IR) was more cell line-specific rather than associated with the cell cycle.

The representative experiments illustrated in Supplementary Figure S17 for GaMG cell line were repeated for each cell line at least 3 times. The results are summarized in Figure 6 as the mean (± SD) values of the degree of DNA damage 30 min and 24 h post-IR in all samples measured under both schedules. As seen in Figure 6, in most cell lines tested (except SW48) the residual damage to DNA after IR and addition of NVP-AUY922 alone or in a combination with PI-103 was much higher than that in drug-free irradiated cells.

As requested by the Referee, we made additional experiments using a much lower radiation dose of 2 Gy (Supplementary Figures S18, S19) and detected the expression of γH2AX. As seen in Supplementary Figures S18, S19, the radiation dose of 2 Gy induced much less DNA damage, which also was repaired much faster, as compared to cells irradiated with 8 Gy (Figure 6). The data are in agreement with the colony-survival curves presented in Figures 1B and 2B.

**Figure 7:**
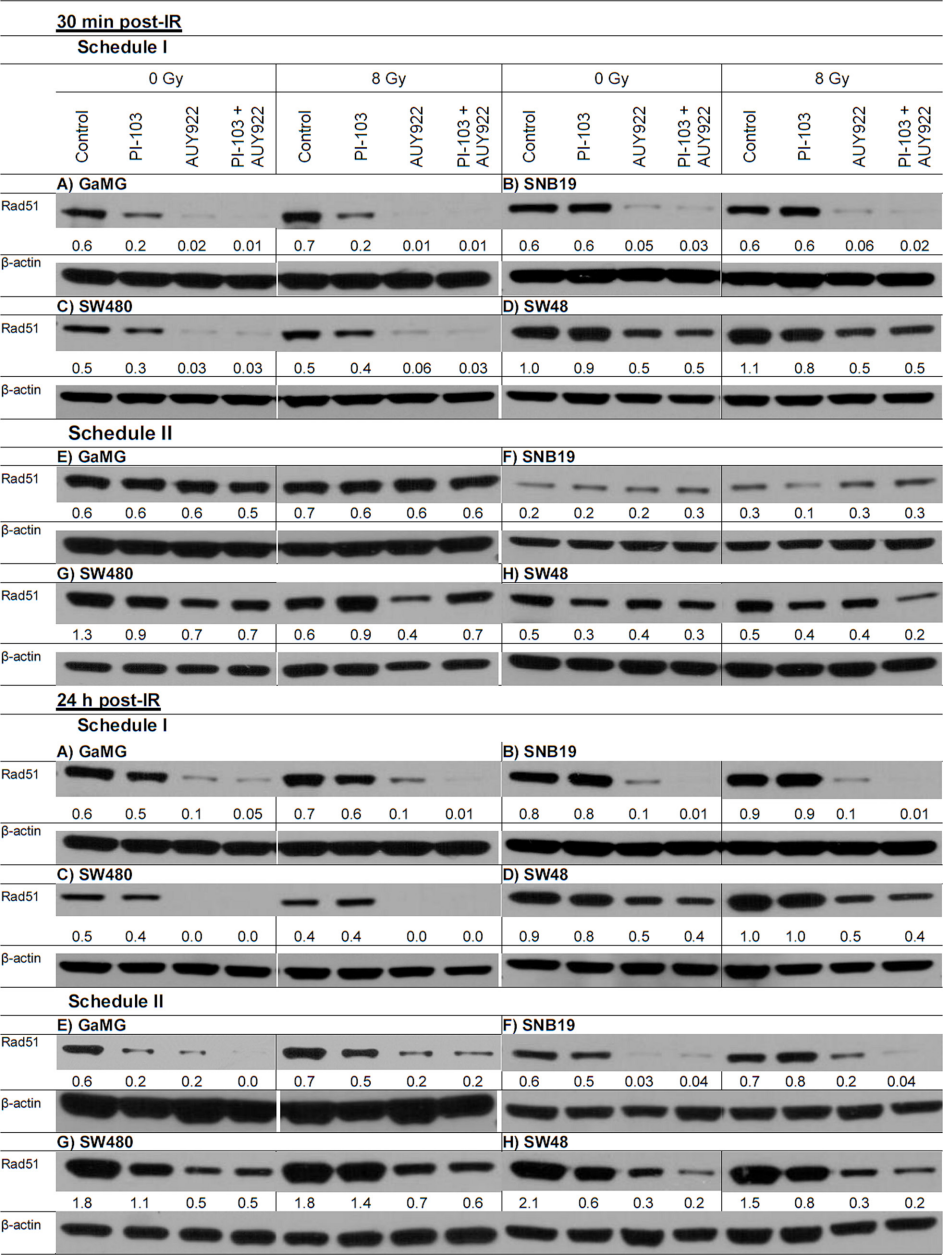
Representative Western blot of the DNA repair protein Rad51 in 4 tumor cell lines subjected to 24-h (Schedule I) or 3-h (Schedule II) pretreatment with the drugs before IR Cell lysates were prepared 30 min and 24 h after IR with 8 Gy. For details, *see* Legend to Figure 3.

Prompted by the observation that both substances given under Schedule II compromised the DNA repair in irradiated cells, we analyzed the expression of the DNA repair protein Rad51. Recently, the expression of this protein has been found to be abrogated by a different PI3K and mTOR inhibitor, NVP-BEZ235 [26, 27]. Figure 7 shows representative Western blot detections of Rad51 protein in 4 cell lines treated with drugs and IR according to both schedules. Thirty minutes after IR, Rad51 was reduced in samples treated with NVP-AUY922 alone or in combination with PI-103 under Schedule I. Twenty four hours post-IR the protein was reduced also under Schedule II after Hsp90 inhibition. In contrast to the published data on NVP-BEZ235 [26, 27], we found that PI-103 alone suppressed the expression of Rad51 only in 2 (GaMG and SW480) out of 4 tested cell lines (Figure 7). This means that the impaired DNA repair capacity revealed by the high residual histone γH2AX levels observed under Schedule II in the majority of cell lines tested cannot be explained by the depletion of Rad51 (Figure 7).

### Effects of PI-103, NVPAUY922 and IR on cell-cycle progression

**Figure 8:**
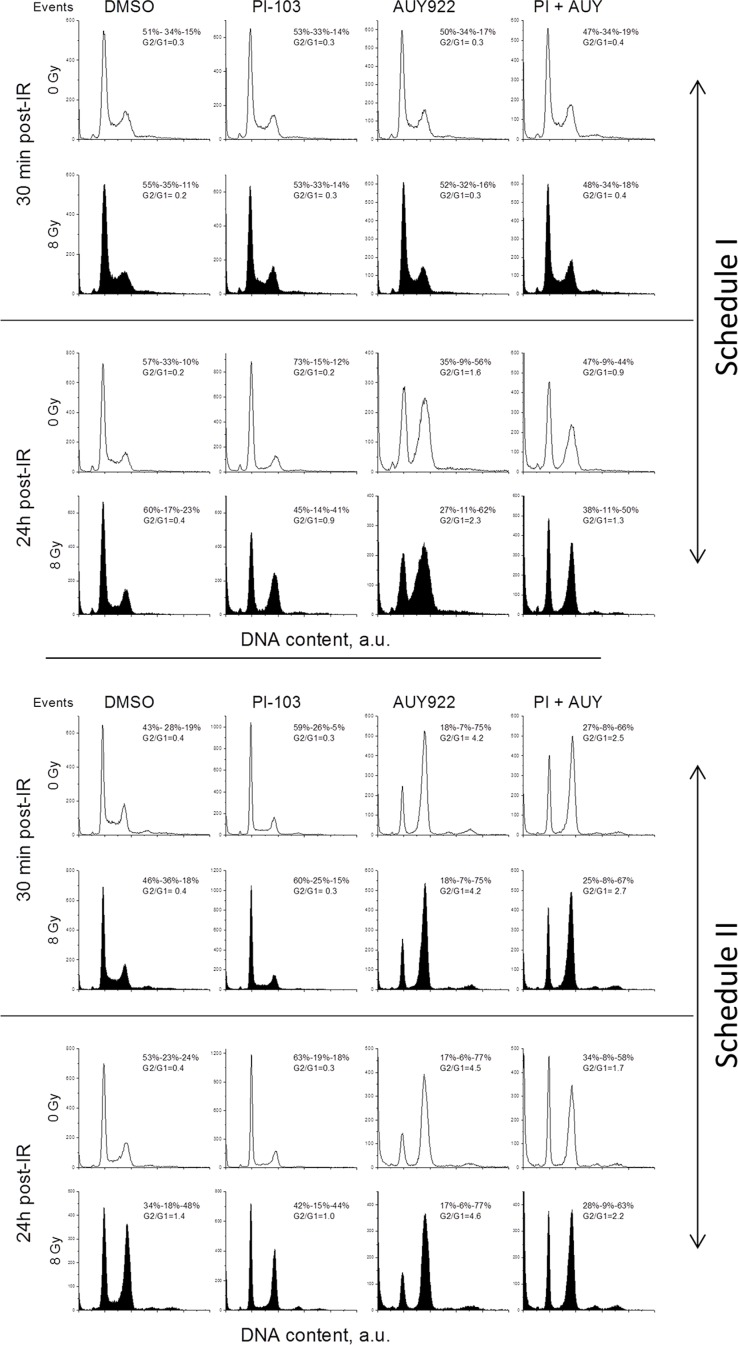
Impact of PI3K/mTOR and Hsp90 inhibitors, IR and combined drug-IR treatment on the cell cycle-phase distribution in GaMG glioblastoma cells Cells were incubated with the substances either 24 h (Schedule I) or 3 h (Schedule II) before IR. After IR with 8 Gy, cells were cultured for 30 min and 24 h, fixed, permeabilized, stained with propidium iodide and analyzed for DNA content by flow cytometry using linear signal amplification. DNA histograms were deconvoluted with ModFit Software. Numbers denoted the percentage of cells in G_1_-, S- and G_2_/M phases and G_2_/G_1_ ratios in each cell sample. Unfilled and filled histograms represent non-irradiated and irradiated samples, respectively. The data are representative of at least three independent experiments. “a.u.” means arbitrary units.

In the following experiments, we analyzed the impact of drugs and IR on the cell-cycle progression as a possible mechanism responsible for the enhanced radiosensitization induced by the combined drug treatment under Schedule II (Figure 2B). Figure 8 shows representative cell cycle histograms for GaMG cells treated under Schedule I (Figure 8 top half) and Schedule II (Figure 8 bottom half), whereas the summarized data for all tested cell lines are shown in Supplementary Figure S20. The large portions of cells in the S- and G_2_/M-phases in non-irradiated control cells in Figure 8 prove that the cell culture was in the exponential growth phase at the beginning of experiments. A 24-h incubation with PI-103 caused an enrichment of G_1_ phase cells from 40–50% to 60–70%. On the contrary, upon a 24-h incubation with the NVP-AUY922, the G_2_/G_1_ ratio increased from 0.4 in control cells to 4.3 (Figure 8, top half) whereas after combined treatment with both inhibitors it was lower (2.5) but still much higher than in control. Thirty minutes post-IR, non-irradiated and irradiated cells (within a particular treatment condition) exhibited similar G_2_/G_1_ ratios (Figure 8, first *versus* second raw). Twenty four hours post-IR, non-treated irradiated GaMG cells showed a G_2_/M arrest (G_2_/G_1_ ratio of 1.4), which was higher than that in the presence of PI-103 (G_2_/G_1_ = 1.0). In the presence of both inhibitors, the G_2_/G_1_ ratio in irradiated cells was about 2.2 which was much lower than that in the presence of NVP-AUY922 alone (4.6).

In case of Schedule II (Figure 8, bottom half), 30 min after IR the cell-cycle histograms of non-treated and drug-treated cells were very similar, independent of IR exposure. A G_2_/M arrest was observed 24 h after IR in the irradiated samples of all cell lines (Supplementary Figure S20). Interestingly, although a 24-h incubation with PI-103 alone caused a strong G_1_ arrest, together with IR under Schedule II, PI-103 caused a massive G_2_/M arrest in the most of tested cell lines and especially in SNB19 cells (Supplementary Figure S20B). As expected, NVP-AUY922 caused S-phase depletion and G_2_/M arrest independent of IR.

To sum up, a 24-h incubation with PI-103 caused a G_1_ arrest, but together with IR the inhibitor produced a massive G_2_/M arrest. A combined treatment with both drugs caused a strong G_2_/M arrest 24 h post-IR in tumor cells irradiated under both schedules, however, to a lesser extent than did Hsp90 inhibition alone.

## DISCUSSION

Clinicians have combined chemotherapy and radiation therapy since the 1980s [28] and the combination of radiation and concurrent chemo- or molecularly targeted therapy has been convincingly shown to be superior to radiation alone in treatment of several cancer forms [29]. A number of anticancer agents including several inhibitors of Hsp90 are known to synergistically enhance the cytotoxicity of IR. Numerous studies, including our own, have already explored Hsp90 as a potential target for tumor cells' radiosensitization [9–11]. A major drawback of Hsp90 inhibition, however, is that it causes up-regulation of Hsp90 itself and his major co-chaperone Hsp70, thereby promoting cell survival by inhibition of caspase-dependent and –independent cell death [13].

The present study aimed to test (*i*) whether preventing the up-regulation of Hsp90 and Hsp70 by PI-103, an inhibitor of PI3K and mTOR, can enhance the radiosensitizing effect of NVP-AUY922 in cancer cell lines, and (*ii*) what is the optimum drug-IR treatment schedule for radiosensitization of tumor cells.

The small synthetic inhibitor of Hsp90, (the isoxazole resorcinol NVP-AUY922), studied here has an improved bioavailability and lower toxicity, the highest affinity for the NH_2_-terminal nucleotide-binding site of Hsp90 [30] as well as beneficial pharmacological properties. It also exhibits strong antiproliferative activity against various tumor cell lines and primary tumors *in vitro* and *in vivo* at well-tolerated doses [31]. We have shown previously that NVP-AUY922 can radiosensitize diverse tumor cell lines [10, 11].

A further substance used in the present study is PI-103, a novel synthetic small molecule of the pyridofuropyrimidine class, which is a potent and selective inhibitor of class I PI3K [32], mTOR and DNA-PK with therapeutic activity against a range of human tumor xenografts [33]. In addition, PI-103 can radiosensitize prostate [34], colorectal [35] and breast [36, 37] cancer cells alone or in a combination with a PARP inhibitor olaparib [37].

In the present study, the two inhibitors were added (alone or in combination) to cells either 24 h before IR. The cells were replated for the colony-forming test immediately after IR (Schedule I), or, alternately, the drugs were added 3 h before IR and the cells were replated 24 h post-IR (Schedule II). Under Schedule I, drug-treated cells were already perturbed before IR with regard to the cell cycle (either G_1_ or G_2_/M arrest) and the activity of the PI3K- and MAPK-pathways, which were undesirably up-regulated in case of PI-103 added alone. In contrast cells treated with PI-103 under Schedule II were not arrested in G_1_ phase during IR exposure. Moreover under Schedule II, the MAPK pathway was not up-regulated yet and, most notably, the PI3K pathway was even strongly repressed. Cells irradiated under Schedule IIs were subjected to further 24 h incubation with the inhibitors before replating for the colony-forming test.

We found that upon long-term (24-h) drug pre-treatment (Schedule I), concomitant PI3K/mTOR inhibition slightly reduced the radiosensitizing activity of the Hsp90 inhibitor in the majority of tested cell lines (Figure 1B). In sharp contrast to the above finding(s), the modified drug-IR treatment schedule, *i.e*. a shorter pretreatment interval of 3 h along with an extended (24 h) post-IR drug exposure (Schedule II), caused a much stronger radiosensitizing effect in 4 tested cell lines than did the Hsp90 inhibitor alone (Figure 2B). Moreover, PI-103 alone acted now as a moderate radiosensitizer in all tested cell lines (Figure 2B). Taken together, the findings presented in Figures 1 and 2 reveal the importance of the treatment schedule for the radiosensitizing activity of PI-103 alone and in combination with NVP-AUY922.

Based on our data, no conclusions can be drawn whether the radiosensitizing effect of PI-103 alone or in a combination with NVP-AUY922 is dependent on the mutational status of *PTEN* or *p53,* because among the four tested cell lines only one was *PTEN* mutated (SNB19), and one (SW48) was *p53* wild type (Figure 2C), yet the effect was observed in all tested cell lines.

In a recent study, PI-103 exhibited a somewhat stronger radiosensitizing effect (enhancement ratio, E.R. = 1.41) in the *PTEN* mutated U251 cells than in the *PTEN* wild-type T98G cells (E.R. = 1.26) [38]. However, the *PTEN* mutated U87-MG line showed in the same study a much lower E.R. of ~1.05 than T98G cells [38]. So a possible correlation between the sensitivity of tumor cells to the PI-103 and their *PTEN* or *p53* status needs further investigation.

In order to elucidate the striking difference in the radiosensitizing effects between two drug-IR schedules reported here (Figure 1B *versus* Figure 2B), we examined the expression of Hsp90/Hsp70, several key proteins of the PI3K and ERK pathways (Figures 3, 4 and Supplementary Figures S3-S16), the degree of apoptosis (Figure 5), induction and repair of DNA damage (Figures 6,7 and Supplementary Figures S17–19), and cell-cycle distribution (Figure 8, Supplementary Figure S20) for either schedule. Based on these data we proposed a simplified model illustrated in Figure 9. In addition, Supplementary Figure S21 summarizes the main differences between both treatment schedules.

**Figure 9:**
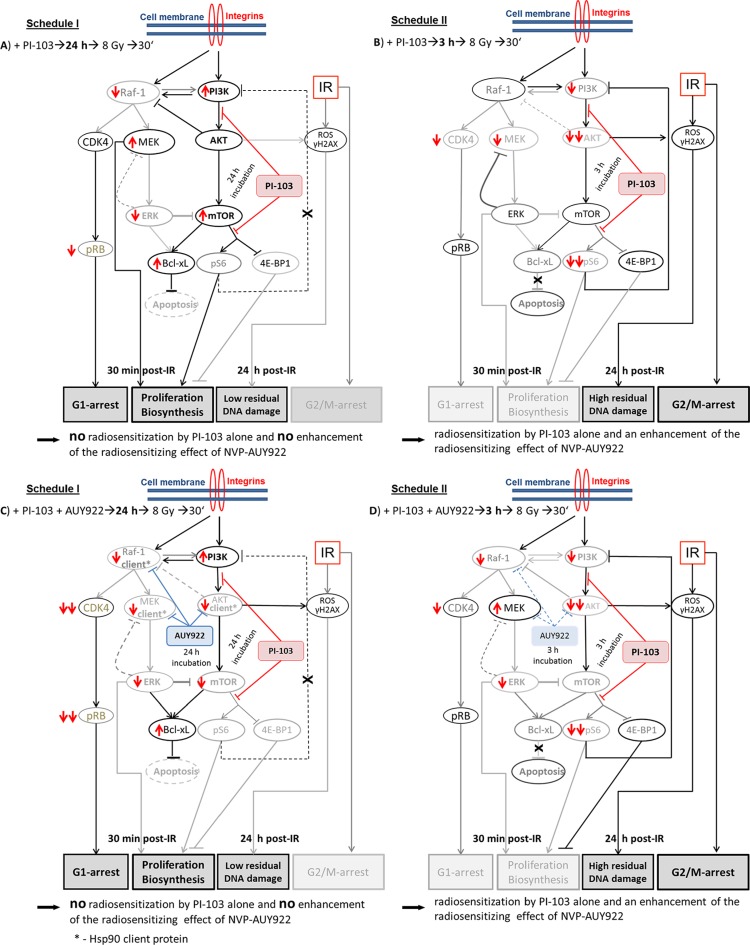
A simplified diagram of putative signaling pathways accountable for the differential responses of tumor cells to PI3K/mTOR- (A-D) and Hsp90- (C, D) inhibition and IR used in two different drug-IR schedules Incubation of tumor cells with PI-103 for 24 h prior to IR (A, C) leads to a reactivation of the PI3K/AKT/mTOR pathway at the time of IR, most likely due to the inhibition of the negative feedback loop mediated by ribosomal protein S6. Subsequently, the activated pro-survival kinase AKT prevents radiation induced apoptosis. Beside this, 30 min after IR we observed the up-regulation of p-ERK1/2 and p-MEK in 3 out of 4 cell lines in the presence of PI-103 used under Schedule I, especially after IR. Raf-1 and p-MEK can be also inhibited through the negative feedback loop mediated by p-ERK [20]. Besides this, the expression of anti-apoptotic Bcl-xL was moderately increased. In contrast, after a short, 3-h incubation with the PI-103, p-AKT is strongly *suppressed* at the time of IR (B, D), which can affect subsequent cell survival. Furthermore, incubation with PI-103 for 24 h (A, C) causes a remarkable arrest in the G_1_-phase. In contrast, cells treated with PI-103 according Schedule II (B, D) showed a strong G_2_/M arrest 24 h after IR. Under Schedule II, drug-treated and irradiated cells showed a delayed DNA damage repair response (Figure 6 and Supplementary Figures S17–S19). To summarize, long-term pre-incubation with PI-103 (A, C) causes no radiosensitization by the substance itself and no enhancement of the radiosensitizing effect of NVP-AUY922 (C). In contrast, a short-term pretreatment with PI-103 (B, D) gives rise to a radiosensitization effect by PI-103 alone and in combination with NVP-AUY922 (D). (Take note of the size of the letters/symbols and the thickness of the lines). For details, *see* text.

Our Western blot analysis revealed that the short-term (3 h) pretreatment with PI-103 caused a depletion of the phosphorylated form of AKT in 2 out of 4 cell lines studied here (Figure 3 RHS and Supplementary Figures S3–S5). In contrast, the long-term (24 h) incubation with the PI-103 led to a reactivation of AKT activity (Figure 3 LHS, and Supplementary Figures S6–S8). The reactivation of AKT suggests the interruption of the negative feedback loops that normally down-regulate PI3K signaling, which in turn can paradoxically promote cell survival, as reported in case of rapalogs (analogs of Rapamycin) elsewhere [39, 40]. Beside this, 30 min after IR we observed the up-regulation of p-ERK1/2 and p-MEK in 3 out of 4 cell lines in the presence of PI-103 used under Schedule I, especially after IR. The activation of ERK-pathway can promote the survival of tumor cells [21, 41], in similar with the activation of PI3K-pathway. The upregulation of both PI3K- and ERK-pathways at the time of IR would explain the lack of radiosensitization by PI-103 used under Schedule I (*i.e*. long-term pretreatment) and the absence of increased tumor cell killing after IR in the presence of both substances (Figure 1B) compared with the action of NVP-AUY922 alone.

A further critical step in the radiation response of cells is apoptosis, which can be regulated by p-AKT, Bcl-xL and other proteins. Our Western blot analysis of the anti-apoptotic protein Bcl-xL revealed that at the time of IR under Schedule I the expression of this protein was moderately increased in most tested cell lines (except SW48) treated with PI-103 or NVP-AUY922, or both (Figure 3 and Supplementary Figures S3–S5).

As mentioned above, the anti-apoptotic protein p-AKT was reactivated (Figure 3 LHS) in PI-103-treated samples after a temporary depletion induced by the short drug application (Figure 3 RHS). Activated AKT is widely recognized as the major mediator of cell survival, which inhibits apoptosis through several mechanisms [42], *e.g*. keeping mitochondrial integrity, phosphorylation and inactivation of the proapoptotic BAD and caspase 9 *etc*. [43]. BAD maintains Bcl-2 and Bcl-xL function, which inhibit apoptosis mainly at the mitochondrial level by suppressing cytochrome *c* release [44]. The activation of Bcl-xL and p-AKT observed here can therefore be responsible, among other factors, for the absence of the increased radiation sensitivity of tumor cells treated with PI-103 alone or in combination with the NVP-AUY922 after long (24 h) preincubation before IR. In contrast, under Schedule II the p-AKT was down-regulated and Bcl-xL was not increased in the samples treated with PI-103 alone or in combination with NVP-AUY922.

Cleaved PARP (Figure 5), an indicator of apoptosis, was observed in the present study only after a 24-h incubation with NVP-AUY922 alone or in combination with PI-103. The lack of apoptosis in cells treated with PI-103 alone is in agreement with the published data [33, 45]. On the other hand, several studies found PI-103-induced apoptosis in radioresistant prostate [34], lung carcinoma [46] and breast [36] cancer cells. Most likely, the induction of apoptosis by PI-103 is cell type-specific [33, 45].

A further critical determinant of radiation-induced cell death is the induction and repair of DNA DSBs, probed in this study by the expression of histone γH2AX (Figure 6 and Supplementary Figures S17–S19). We found that the kinetics of DNA damage repair differed markedly between two treatments protocols. In cells pretreated with NVP-AUY922 alone or in a combination with PI-103 under Schedule I, the DNA damage recovered much faster than in respective samples treated according Schedule II, which showed an elevated residual DNA damage levels 24 h after IR. This finding corroborates the results of Kao and co-workers [47] who showed that inhibition of PI3K activity probably does not abolish the sensing of DNA damage but rather leads to a reduced repair of this damage.

Next we found a cell type-specific reduction of DNA repair protein Rad51 after PI-103 treatment and an almost complete disappearance of Rad51 after prolonged Hsp90 inhibition (Figure 7). The reason for the inhibition of Rad51 by PI-103 can be the drug-mediated G_1_ arrest, similar to that reported for a different PI3K and mTOR inhibitor NVP-BEZ35 [27, 48]. It is known that Rad51 operates mostly during Homologous Recombination (HR) of DNA, which appears to be active only from the mid-S to G_2_ phases of the cell cycle [49]. Accordingly, a reduced portion of cells in these cell cycle phases might have led to a decline in Rad51 expression. This result was also confirmed in our experiments. Another finding is that prolonged treatment with the Hsp90 inhibitor lead to a complete loss of Rad51, as seen in Figure 7, which is in accordance with the published literature [50–52] showing that Hsp90 inhibition by 17-AAG down-regulates Rad51 in NSCLC [52] and prostate [50] carcinoma cells. Similarly, NVP-AUY922 abrogates HR in head and neck squamous carcinoma cells [51]. At the same time, the expression patterns of Rad51 were similar under both treatment schedules (Figure 7). Accordingly, Rad51 cannot be responsible for the impaired DNA repair observed under Schedule II.

In addition to the above considerations, the differences in cells' radiosensitivity treated with PI-103 alone or in a combination with NVP-AUY922 between both schedules (Figures 1B, 2B) can be partly explained by the peculiarities of cell cycle distribution. Thus, long-term treatment with PI-103 (Schedule I) prior to IR caused the cells to reside predominantly in G_1_ phase (Figure 8 and Supplementary Figure S20), which is the most radioresistant cell cycle phase. Interestingly, combined PI-103-IR treatment under Schedule II caused a strong G_2_/M block 24 h after IR (Figure 8 and Supplementary Figure S20). As a result, cells in the G_2_/M phase may be more radiosensitive than those treated with PI-103 alone and irradiated under Schedule I.

Taken together, the results presented here provide clear evidence for the importance of drug-IR schedule. Our results are in line with findings that the efficacy of the combined radiochemotherapy relies, among others factors, on the schedule of drug administration [28, 29]. Particularly, the combination of gemcitabine followed by gefitinib (an inhibitor of the epidermal growth factor receptor) has been found be more effective in controlling tumor growth than the reverse drug schedule [29].

To sum up, our data demonstrate an enhanced radiosensitivity in tumor cells pretreated simultaneously with PI3K/mTOR and Hsp90 inhibitors shortly before IR compared with the cells treated with Hsp90 inhibitor alone. The complex mechanisms underlying the increased radiosensitization by PI-103 and NVP-AUY922 inhibitors involve apparently several, cell line-specific pathways that lead to the down-regulation of PI3K- and ERK-pathways at the moment of IR, strong G_2_/M arrest and protracted DNA damage repair 24 h thereafter, and to a lesser extent, to apoptosis. In contrast, the long-term treatment with PI-103 before IR caused a reactivation of PI3K and MAPK prosurvival pathways, G_1_ arrest at the moment of IR, and a proficient DNA damage repair. Yet the observed strong arrest of tumor cells in G_1_ phase justified the use of the PI-103 as a potential cytostatic drug. Finally, our *in vitro* data reveal the importance of the duration of PI3K/mTOR inhibition before IR for the radiosensitization of tumor cells by Hsp90 inhibitors and pave the way for future exploration of combination of molecularly targeted therapy and radiation on *in vivo* mouse tumor model. The data are of particular interest for the cancer therapy, because NVP-AUY922 is currently in clinical trials Phase I-II (*www.clinicaltrials.gov*), but without IR yet. Besides raising important questions concerning the mechanisms of radiosensitization, the *in vitro* data presented here will surely prompt further clinical studies on the possibility of combining NVP-AUY922 and PI-103 with radiation, which may open up a promising approach for improved local control of cancer.

## MATERIALS AND METHODS

### Cells

The set of human tumor cell lines studied here includes 2 glioblastoma, GaMG (*PTEN* wt, *p53* mut), and SNB19 (*PTEN* mut, *p53* mut), and 2 colon carcinoma, SW480 (*PTEN* wt, *p53* mut, *kRas* mut), and SW48 (*PTEN* wt, *p53* wt) cell lines. All cell lines were obtained from the American Type Culture Collection (Manassas, VA) and routinely cultured under standard conditions (5% CO_2_, 37°C) in complete growth medium (CGM), which was either DMEM (GaMG, SNB19) or RPMI-1640 (SW480, SW48), supplemented with 10% fetal bovine serum.

### Cell ciability assay

The intracellular ATP level, as an indicator of cell viability, was determined by means of the CellTiter-Glo Luminiscent Cell Viability Assay (Promega, Madison, WI) according to the manufacturer's instructions. Serial dilutions of PI-103 (0–20 μM) in CGM were added to cell cultures in quadruplicates and the drug cytotoxicity was determined 24 h later. The concentration of NVP-AUY922 was kept constant at 200 nM [10]. Control samples contained the respective concentrations of DMSO.

### Drug treatment

Both drugs were obtained from Selleckchem (Absource Diagnostics GmbH, Munich, Germany). The drugs were freshly diluted from frozen aliquots in DMSO stored at 20°C. PI-103 (2 μM, [15]) and NVP-AUY922 (200 nM, [10]) were applied in two different schedules (Supplementary Figure S2). In Schedule I the substances were added 24 h before IR and kept during and after IR. Under Schedule II the drugs were added 3 h prior to IR and remained in CGM up to 24 h post-IR. Cells treated in parallel with respective amounts of DMSO served as controls.

### Antibodies

The primary and secondary antibodies are specified in Supplementary Information.

### X-ray irradiation

Irradiation was performed at room temperature using a 6 MV Siemens linear accelerator (Siemens, Concord, CA) at a dose rate of 2 Gy/min. After irradiation, cells were kept in CGM for the indicated time until harvest.

### Colony survival assay

Cell survival curves were generated by a standard colony formation assay as previously described. [53] Subconfluent monolayers of drug-treated and non-treated cells were irradiated in culture flasks filled with CGM at room temperature by graded single doses (0 8 Gy), seeded either 30 min (Schedule I) or 24 h (Schedule II) post-IR in Petri dishes and then cultivated in CGM for the next 2 weeks. After 2 weeks, the cells were fixed and stained with crystal violet (0.6%). The mean survival data for each individual cell line were fitted to the linear quadratic model (LQ, Equation 1):

*SF* = exp (−*αX* − *βX*^2^) (Equation 1),

where, *SF* is the survival fraction, *X* is the irradiation dose, *α* and *β* are the fitted parameters.

### Western blot

For immunoblot analysis, whole-cell lysates were prepared according to standard procedures. Samples equivalent to 20 40 μg of protein were separated using 4–12% SDS-polyacrylamide pre-cast gels (Invitrogen, Karlsruhe, Germany) and transferred to nitrocellulose membranes. For protein detection, membranes were incubated with respective primary and species-specific peroxidase-labeled secondary antibodies according to standard protocols. The levels of protein expression were quantified using the ImageJ software (NIH, Bethesda, MD) and normalized to the β-actin levels.

### Detection of histone γH2AX and cell-cycle measurements by flow cytometry

Nontreated and drugtreated cell cultures were irradiated as subconfluent monolayers in CGM at room temperature. The cells were then incubated under standard conditions and analyzed by flow cytometry 30 min and 24 h after IR exposure. For analysis, cells were trypsinized, washed twice in PBS, fixed and stained for γH2AX according to a protocol described elsewhere [54]. The cells were then counterstained with propidium iodide (Sigma P-4170, 10 μg/ml) in the presence of ribonuclease A (Sigma R-5250, 25 μg/ml) as described elsewhere [55]. At least 20,000 cells were assayed for histone γH2AX and DNA distribution using a flow cytometer FACSCantoII (Becton Dickinson, San Jose, CA). Cellular green (histone γH2AX) or red fluorescence (Propidium iodide-DNA) was acquired in logarithmic or linear mode, respectively. The output data presented as one-dimensional histograms, *i.e*. the distributions of histone γH2AX or Propidium iodide-DNA signals within cell samples, were analyzed using the Flowing Software program obtained from P. Terho (Turku Centre for Biotechnology, Turku, Finland) and the ModFit LT program (Verity Software House, Topsham, ME).

### Statistics

Data are presented as means (± SD or ± SE). Mean values were compared by the Student's *t*-test. The threshold of statistical significance was set at *P* < 0.05. Statistics and fitting of experimental data were performed with Origin 8.5 (Microcal, Northampton, MA).

## SUPPLEMENTARY MATERIALS AND METHODS, FIGURES AND TABLES


