# Supplementary material for: Dual PI3K- and mTOR-inhibitor PI-103 can either enhance or reduce the radiosensitizing effect of the Hsp90 inhibitor NVP-AUY922 in tumor cells: The role of drug-irradiation schedule
Source: Oncotarget. 2016 May 20;7(25):38191–209. doi: 10.18632/oncotarget.9501 (PMC5122382; doi:10.18632/oncotarget.9501)
Supplement: Supplementary file 1 [file oncotarget-07-38191-s001.pdf]

Dual PI3K- and mTOR-inhibitor PI-103 can either enhance or reduce the radiosensitizing effect of the Hsp90 inhibitor NVP-AUY922 in tumor cells: The role of drug-irradiation schedule

Supplementary Materials

MATERIALS AND METHODS

Antibodies

The primary antibodies used were: rabbit polyclonal anti-PTEN, rabbit polyclonal anti-PI3K p110, mouse monoclonal anti-phospho-AKT (Ser473), rabbit polyclonal anti-AKT, rabbit monoclonal anti-phospho-mTOR (Ser2448), rabbit polyclonal anti-mTOR, rabbit polyclonal anti-phospho-S6 (Ser240/244), mouse monoclonal anti-S6 ribosomal protein (54D2), mouse monoclonal anti-phospho-4E BP1, rabbit polyclonal anti-Bcl-xL, rabbit polyclonal anti-phospho-Rb (Ser780), mouse monoclonal anti-cdk1 (POH1), rabbit monoclonal anti-phospho-MEK1/2 (Ser217/221), MEK1/2 rabbit monoclonal anti-phospho-p44/42 MAPK (ERK1/2) (Thr202/Tyr204), rabbit anti-p44/42 MAPK (ERK1/2), rabbit monoclonal anti-Rad51, rabbit polyclonal anti-PARP (all from Cell Signaling, Danvers, MA), rabbit polyclonal anti-cdk4, rabbit polyclonal anti-Raf-1, goat anti-4E-BP1 (Santa Cruz, Dallas, TX), mouse anti-p53 (Merck, Darmstadt, Germany), mouse monoclonal anti-Hsp70, mouse monoclonal anti-Hsp90 (BD, Heidelberg, Germany) mouse monoclonal anti-β-actin (Sigma, Deisenhofen, Germany) and mouse monoclonal anti-phospho-histone H2AX (Ser139) FITC-conjugate (Millipore, Schwalbach, Germany). Secondary species-specific antibodies for western blot were labelled with horseradish-peroxidase (DAKO, Hamburg, Germany).

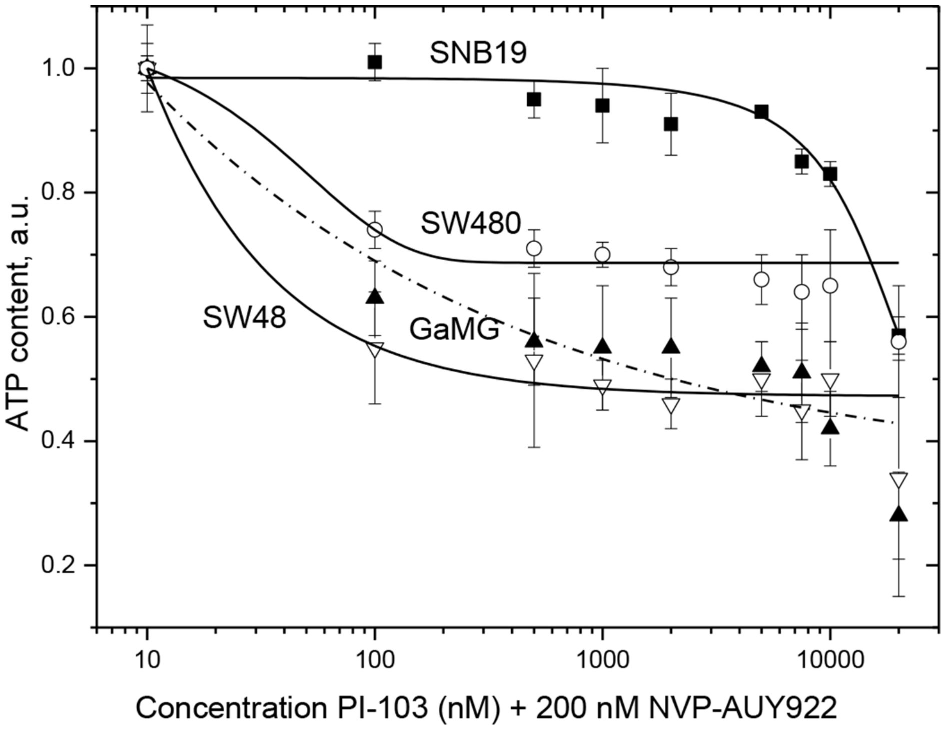

Supplementary Figure S1: Cellular viability measured by an ATP test. Changes of intracellular ATP in 4 tumor cells exposed to serial dilutions of PI-103 in the presence of 200 nM of NVP-AUY922 for 24 hours. ATP content was measured by standard luciferase bioluminescence assay. Quadruplicate data derived from at least three independent experiments were averaged, normalized against non-treated controls (DMSO) and analyzed using the standard four-parameter logistic model to generate dose-response curves. Error bars indicate SD values. “a.u.” means arbitrary units.

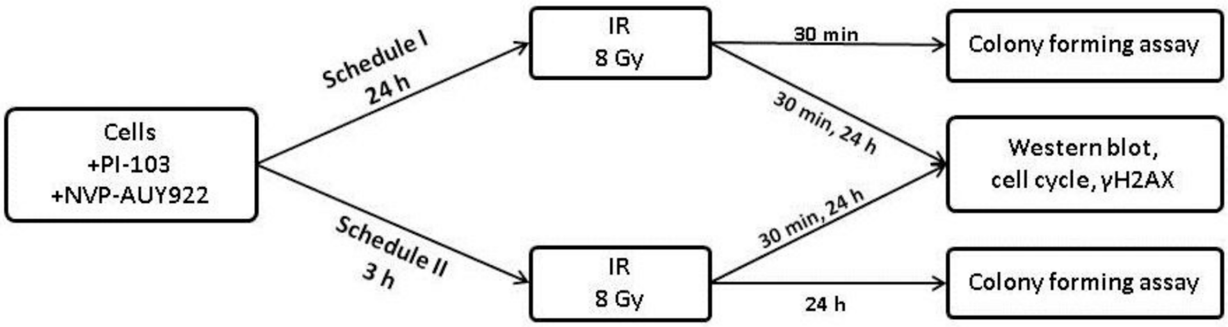

Supplementary Figure S2: Two different drug-irradiation (IR) schedules were used in the study. PI-103 and NVP-AUY922 were added to the tumor cell cultures either for 24 h (Schedule I) or 3 h (Schedule II) before IR and kept in culture medium up to 24 h post-IR. Cells treated in different schedules were then analyzed at indicated times by colony-forming ability, expression of marker proteins, DNA damage and cell-cycle progression.

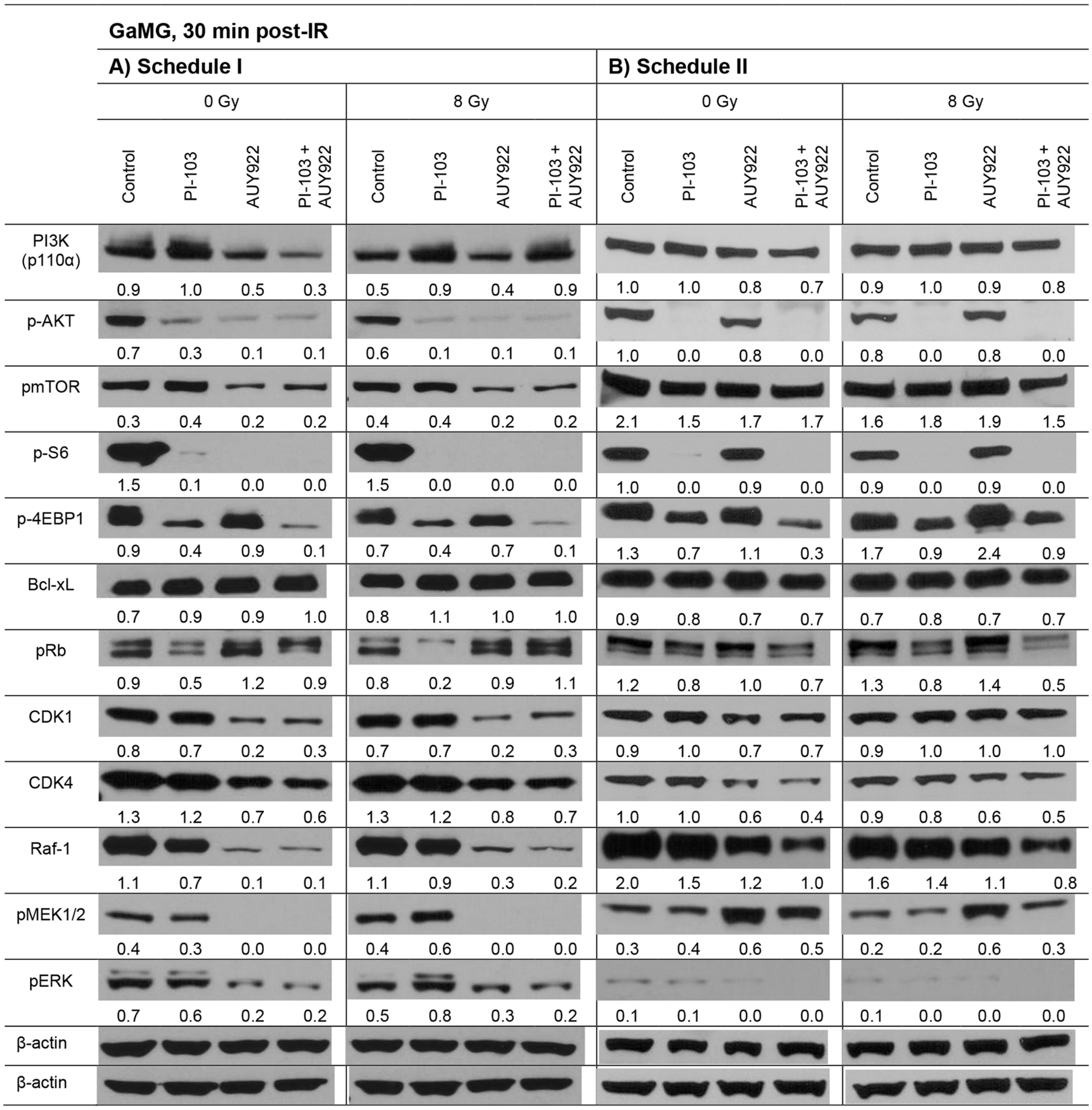

Supplementary Figure S3: Effects of PI-103, NVP-AUY922 and IR on the expression levels of marker proteins in GaMG cell line detected 30 min post-IR. Cells were treated with PI-103, NVP-AUY922 and IR either in Schedule I (**A**) or Schedule II (**B**). For details, *see* Legend to Figure 3.

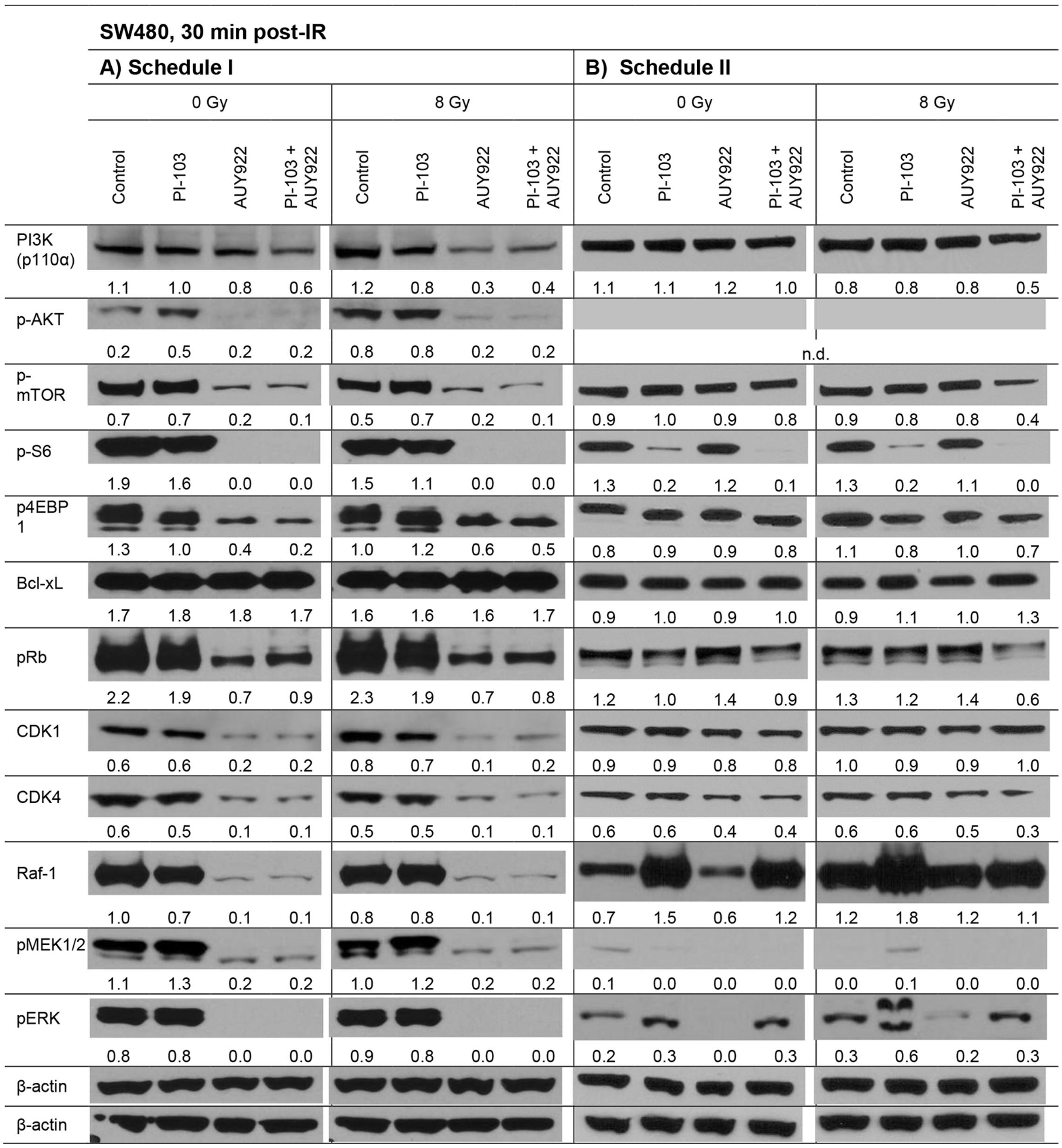

Supplementary Figure S4: Effects of PI-103, NVP-AUY922 and IR on the expression levels of marker proteins in SW480 cell line detected 30 min post-IR. Cells were treated with PI-103, NVP-AUY922 and IR either in Schedule I (**A**) or Schedule II (**B**). For details, *see* Legend to Figure 3. “n.d.” indicates not determined.

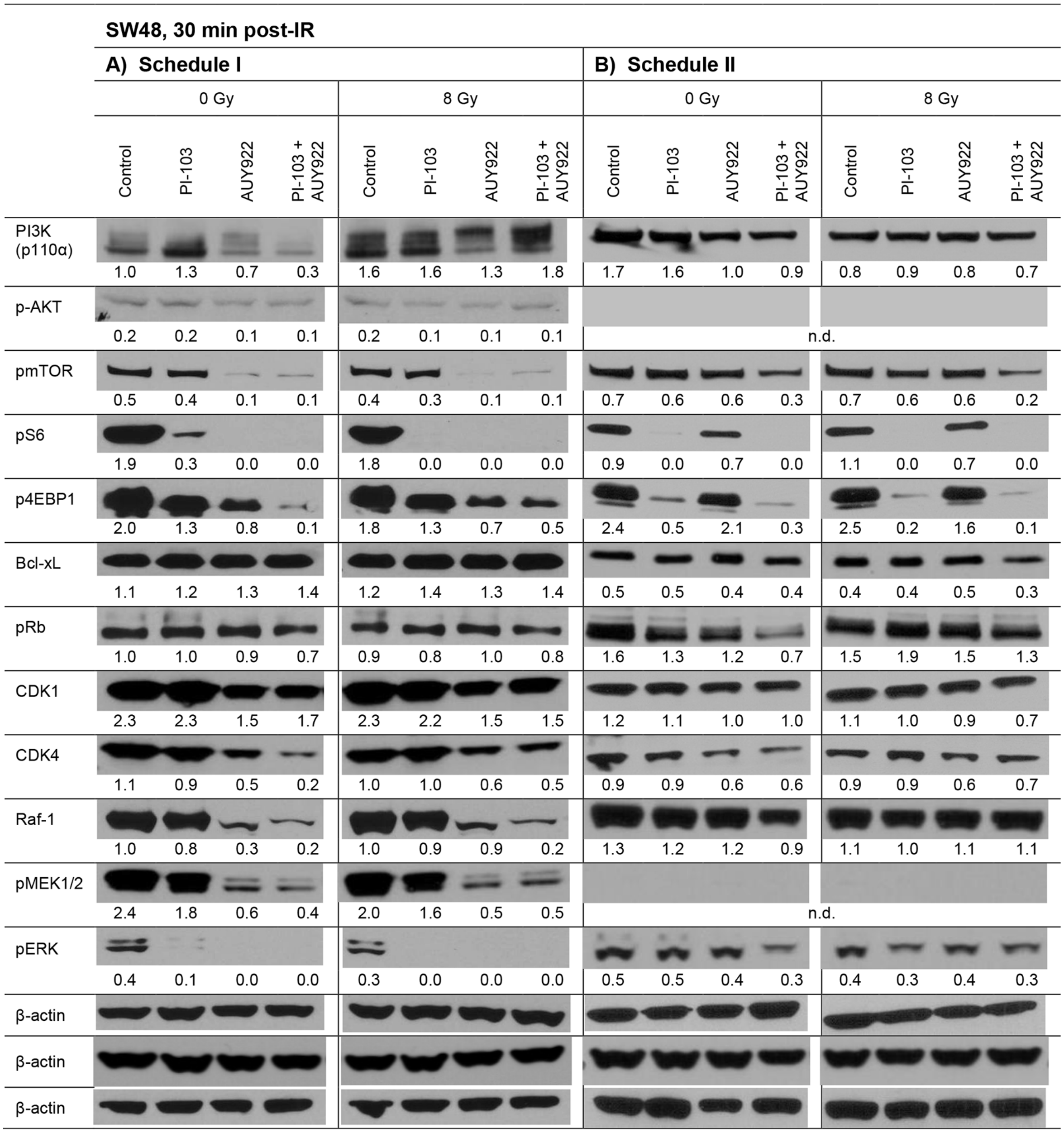

Supplementary Figure S5: Effects of PI-103, NVP-AUY922 and IR on the expression levels of marker proteins in SW48 cell line detected 30 min post-IR. Cells were treated with PI-103, NVP-AUY922 and IR either in Schedule I (**A**) or Schedule II (**B**). For details, *see* Legend to Figure 3. “n.d.” indicates not determined.

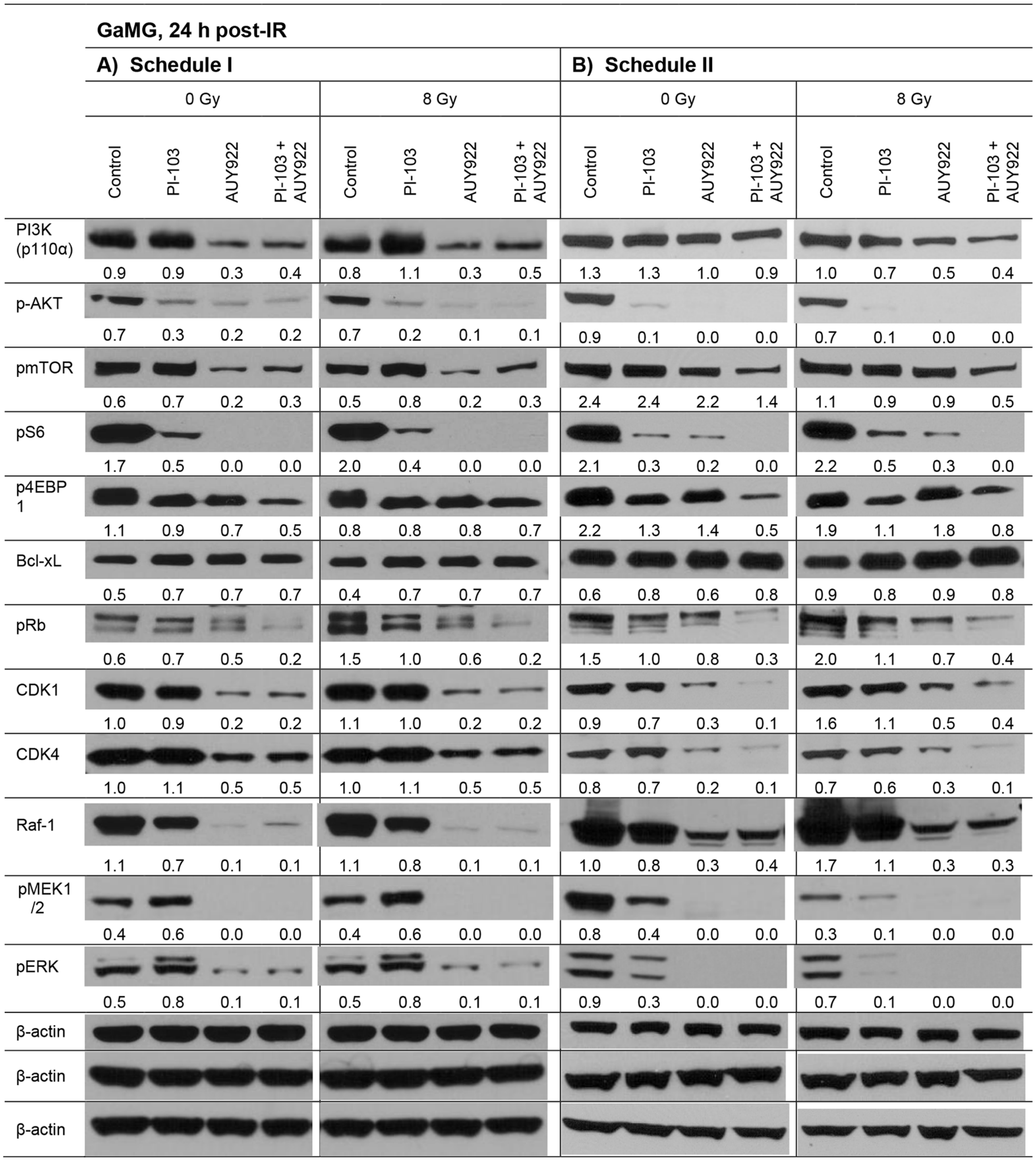

Supplementary Figure S6: Effects of PI-103, NVP-AUY922 and IR on the expression levels of marker proteins in GaMG cell line detected 24 h post-IR. Cells were treated with PI-103, NVP-AUY922 and IR either in Schedule I (**A**) or Schedule II (**B**). For details, *see* Legend to Figure 3.

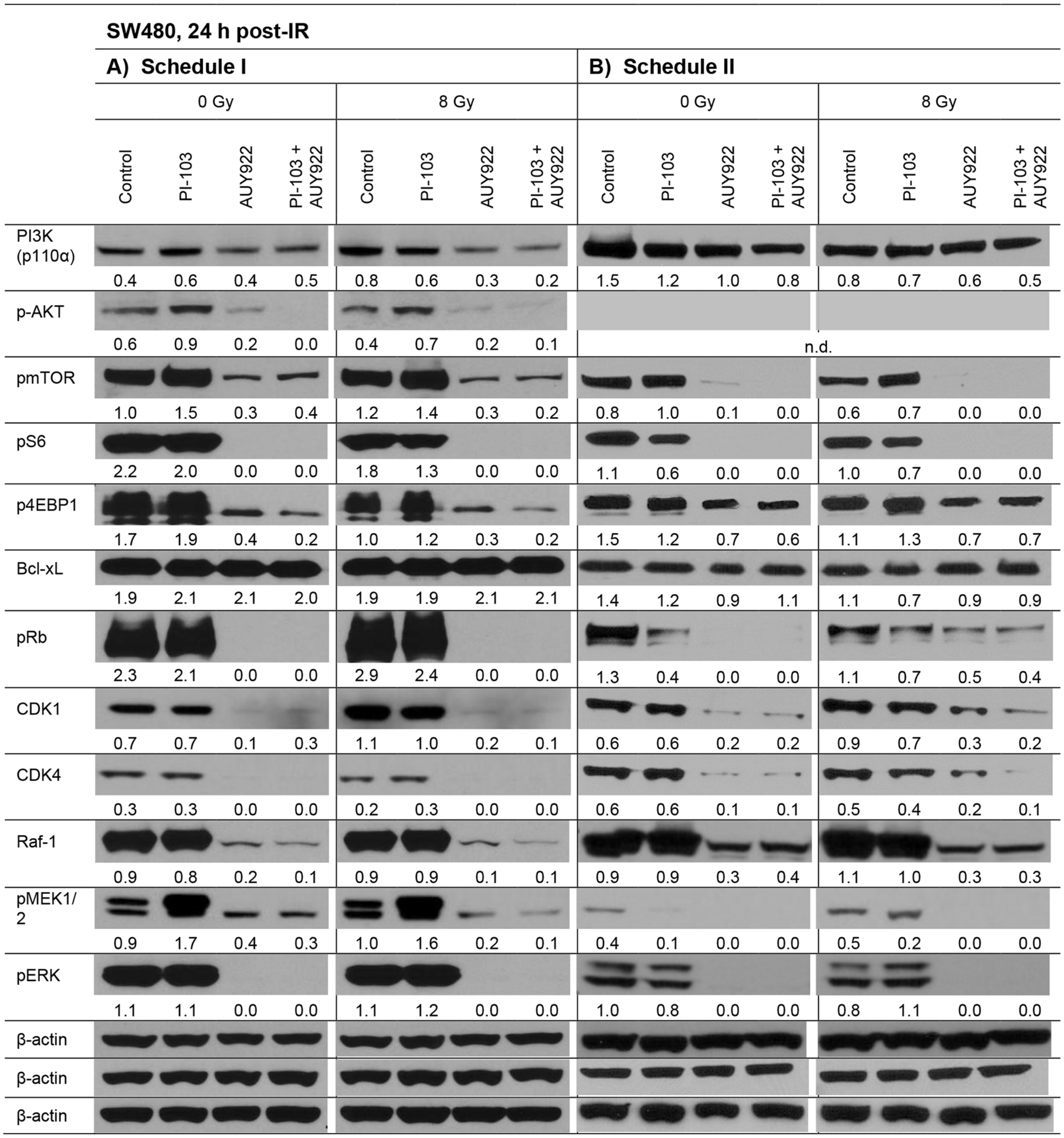

Supplementary Figure S7: Effects of PI-103, NVP-AUY922 and IR on the expression levels of marker proteins in SW480 cell line detected 24 h post-IR. Cells were treated with PI-103, NVP-AUY922 and IR either in Schedule I (**A**) or Schedule II (**B**). For details, *see* Legend to Figure 3. “n.d.” indicates not determined.

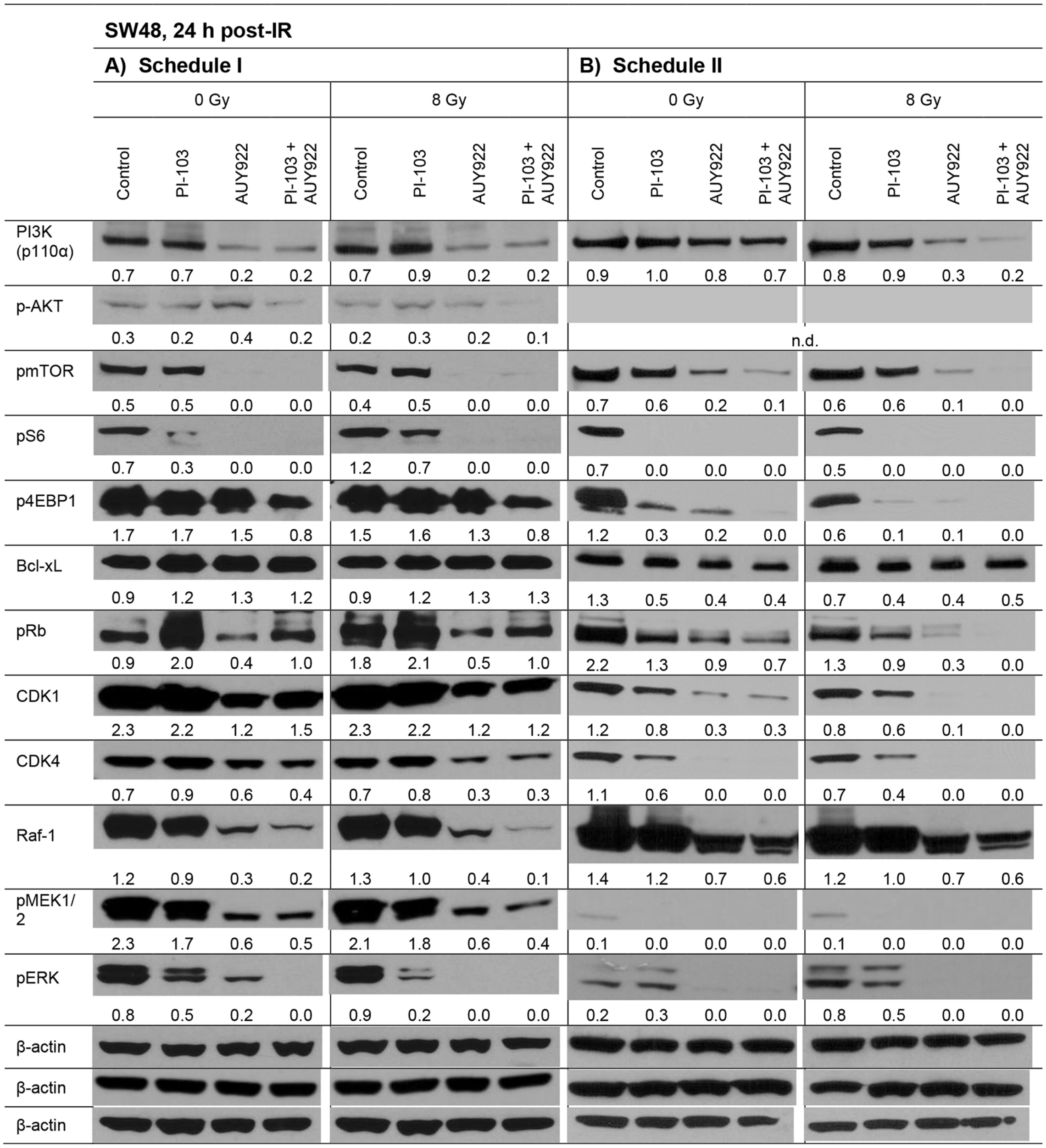

Supplementary Figure S8: Effects of PI-103, NVP-AUY922 and IR on the expression levels of marker proteins in SW48 cell line detected 24 h post-IR. Cells were treated with PI-103, NVP-AUY922 and IR either in schedule I (**A**) or schedule II (**B**). For details, *see* legend to Figure 3. “n.d.” indicates not determined.

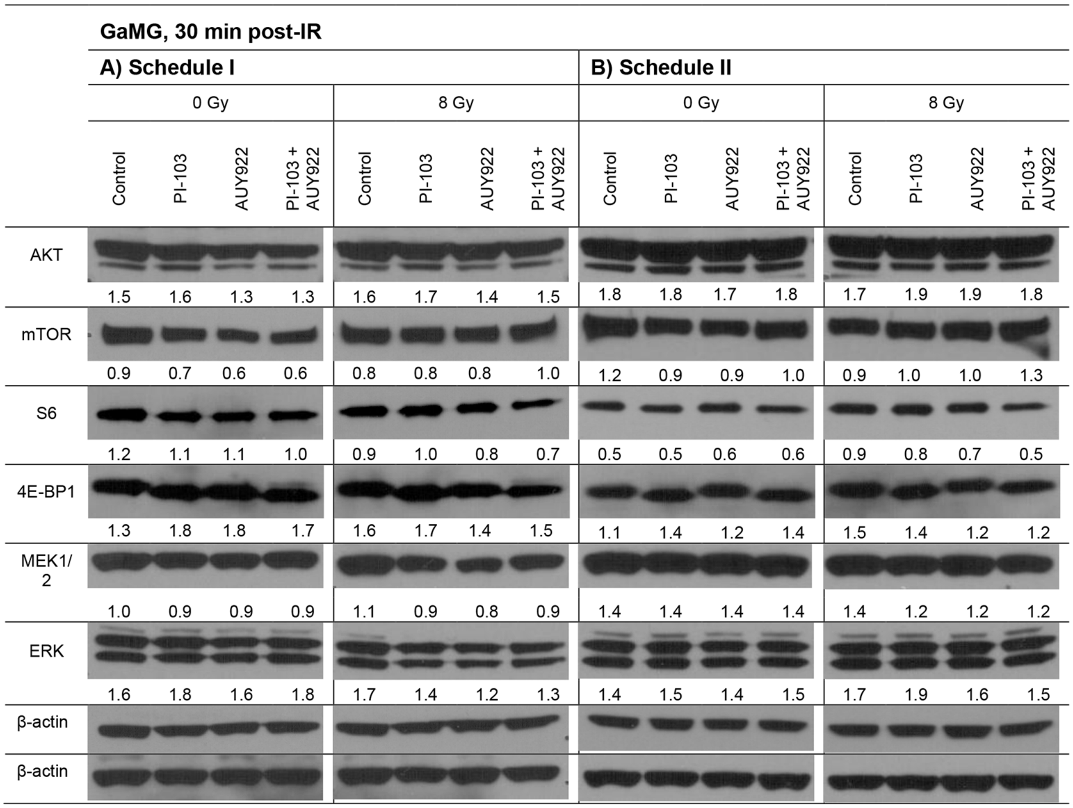

Supplementary Figure S9: Effects of PI-103, NVP-AUY922 and IR on the expression levels of marker proteins in GaMG cell line detected 30 min post-IR. Cells were treated with PI-103, NVP-AUY922 and IR either in Schedule I (**A**) or Schedule II (**B**). For details, *see* Legend to Figure 3.

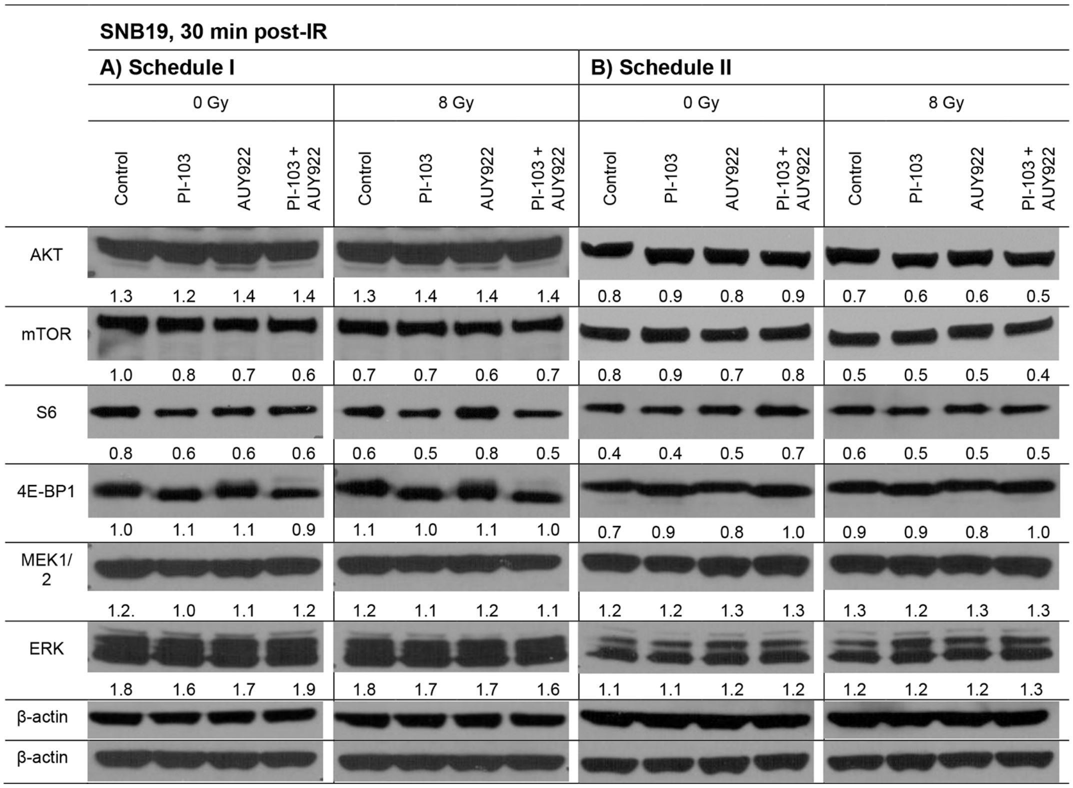

Supplementary Figure S10: Effects of PI-103, NVP-AUY922 and IR on the expression levels of marker proteins in SNB19 cell line detected 30 min post-IR. Cells were treated with PI-103, NVP-AUY922 and IR either in Schedule I (**A**) or Schedule II (**B**). For details, *see* Legend to Figure 3.

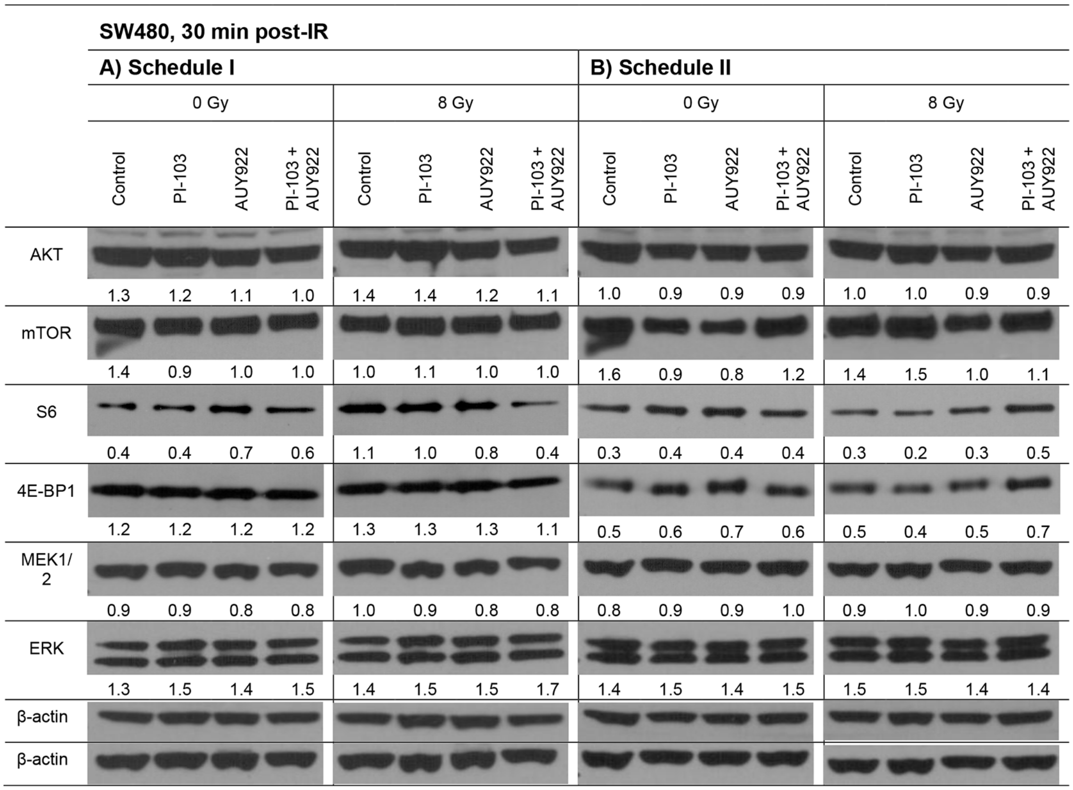

Supplementary Figure S11: Effects of PI-103, NVP-AUY922 and IR on the expression levels of marker proteins in SW480 cell line detected 30 min post-IR. Cells were treated with PI-103, NVP-AUY922 and IR either in Schedule I (**A**) or Schedule II (**B**). For details, *see* Legend to Figure 3.

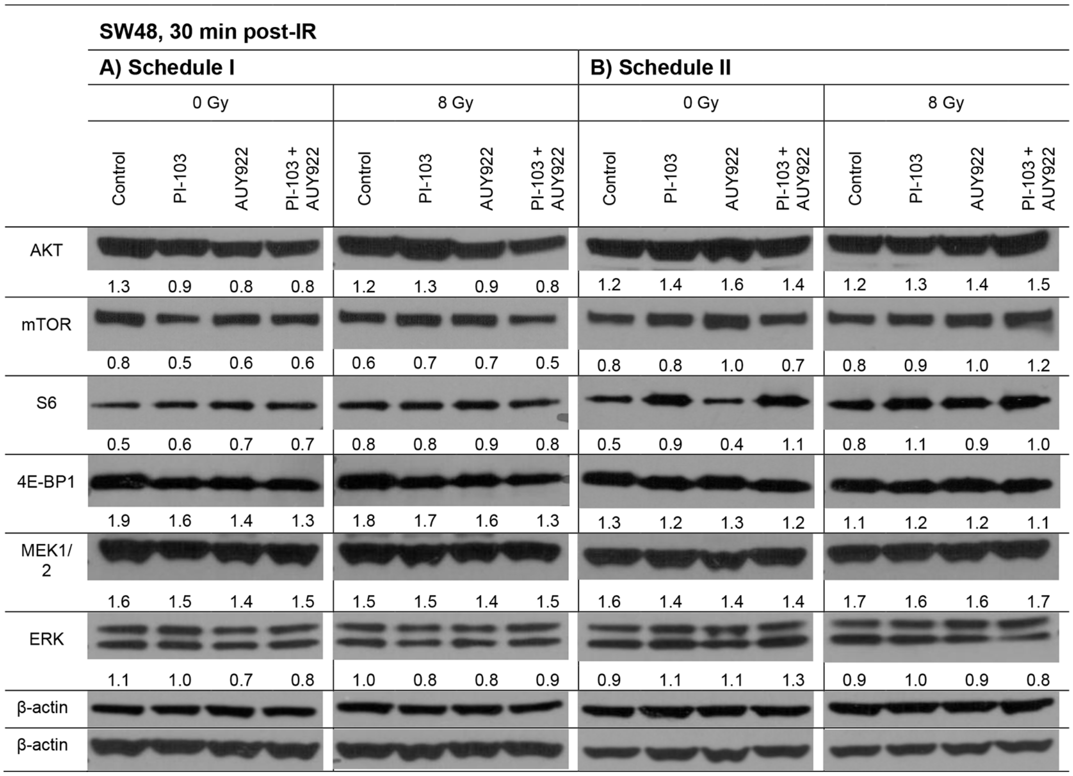

Supplementary Figure S12: Effects of PI-103, NVP-AUY922 and IR on the expression levels of marker proteins in SW48 cell line detected 30 min post-IR. Cells were treated with PI-103, NVP-AUY922 and IR either in Schedule I (**A**) or Schedule II (**B**). For details, *see* Legend to Figure 3.

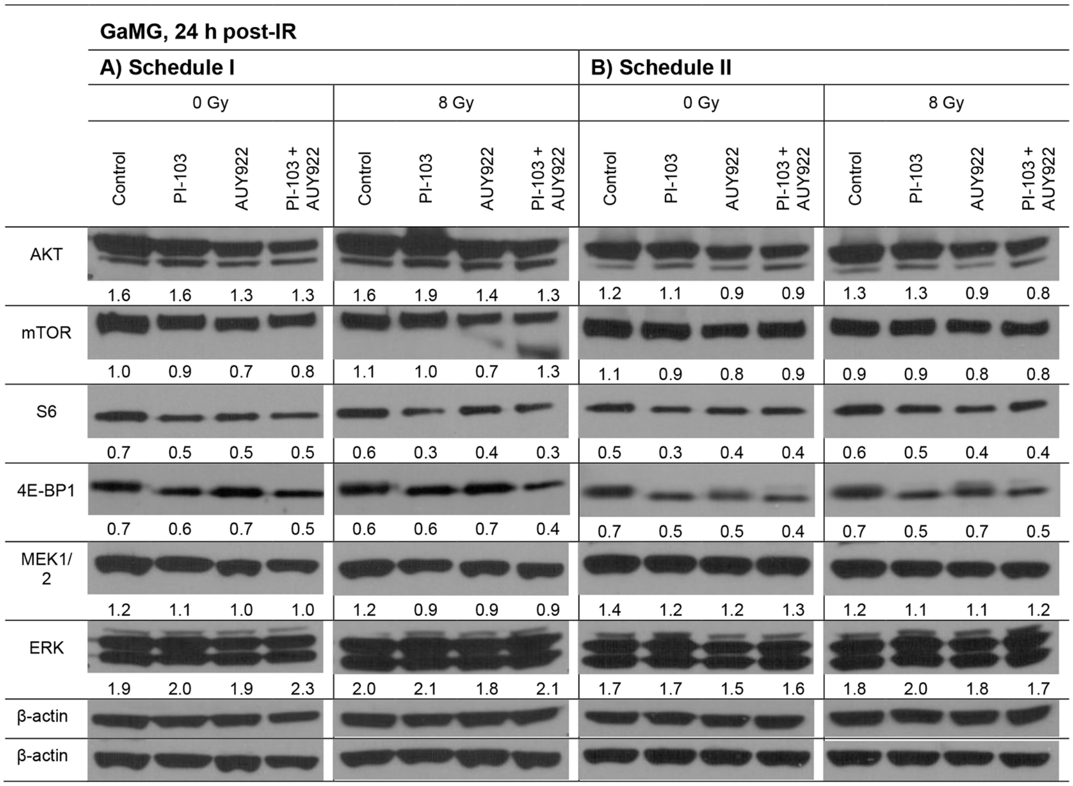

Supplementary Figure S13: Effects of PI-103, NVP-AUY922 and IR on the expression levels of marker proteins in GaMG cell line detected 24 min post-IR. Cells were treated with PI-103, NVP-AUY922 and IR either in Schedule I (**A**) or Schedule II (**B**). For details, *see* Legend to Figure 3.

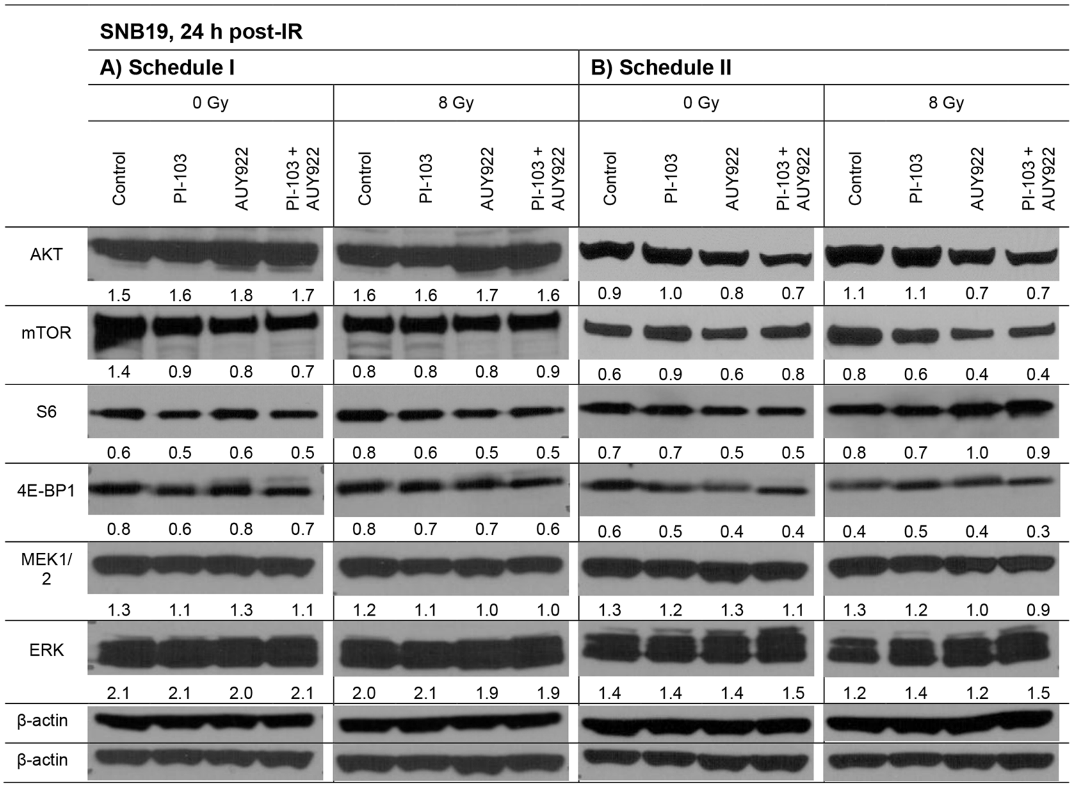

Supplementary Figure S14: Effects of PI-103, NVP-AUY922 and IR on the expression levels of marker proteins in SNB19 cell line detected 24 h post-IR. Cells were treated with PI-103, NVP-AUY922 and IR either in Schedule I (**A**) or Schedule II (**B**). For details, *see* Legend to Figure 3.

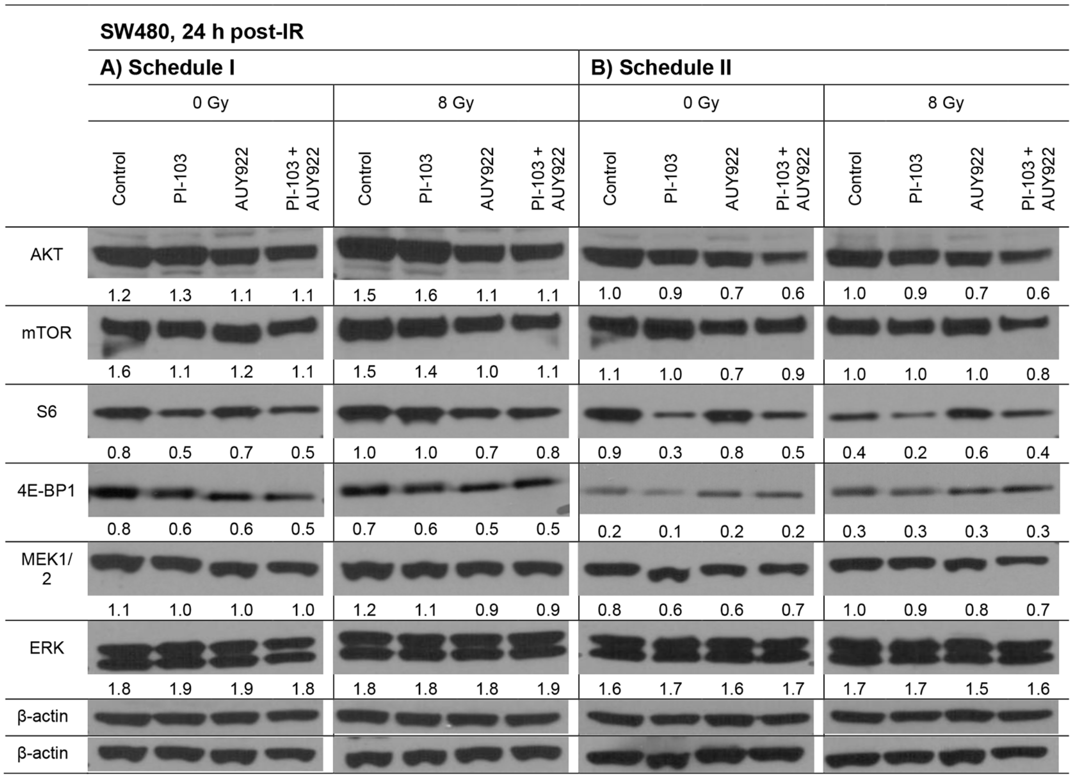

Supplementary Figure S15: Effects of PI-103, NVP-AUY922 and IR on the expression levels of marker proteins in SW480 cell line detected 24 h post-IR. Cells were treated with PI-103, NVP-AUY922 and IR either in Schedule I (**A**) or Schedule II (**B**). For details, *see* Legend to Figure 3.

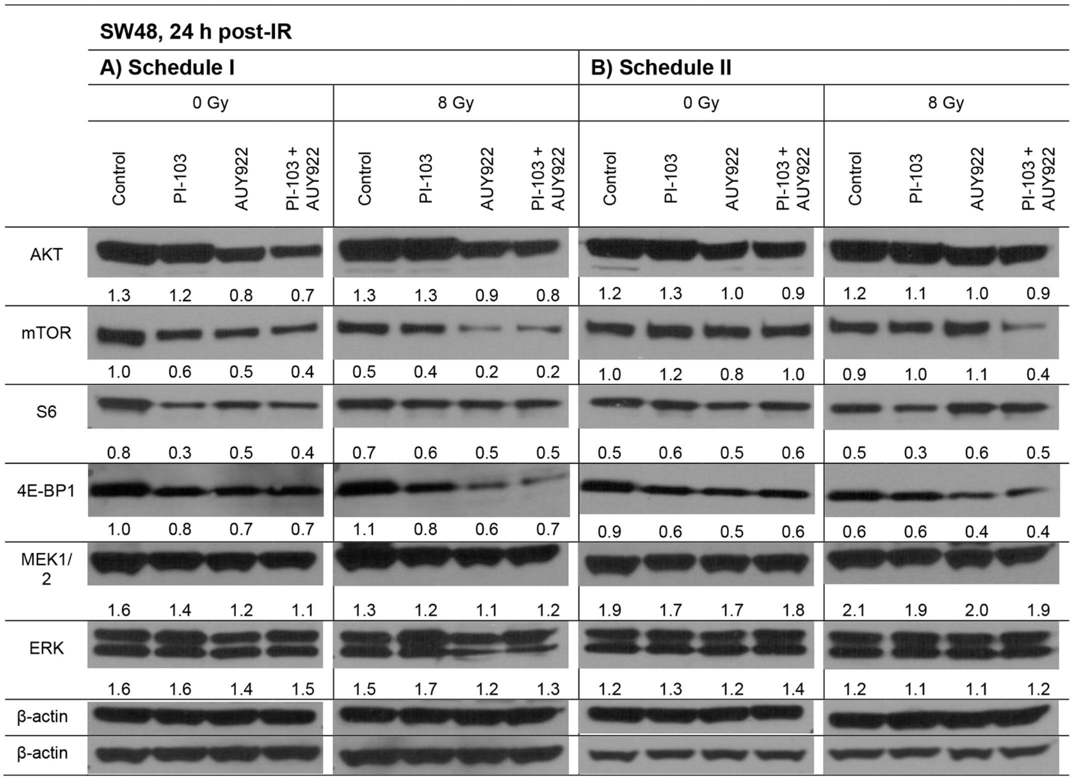

Supplementary Figure S16: Effects of PI-103, NVP-AUY922 and IR on the expression levels of marker proteins in SW48 cell line detected 24 h post-IR. Cells were treated with PI-103, NVP-AUY922 and IR either in Schedule I (**A**) or Schedule II (**B**). For details, *see* Legend to Figure 3.

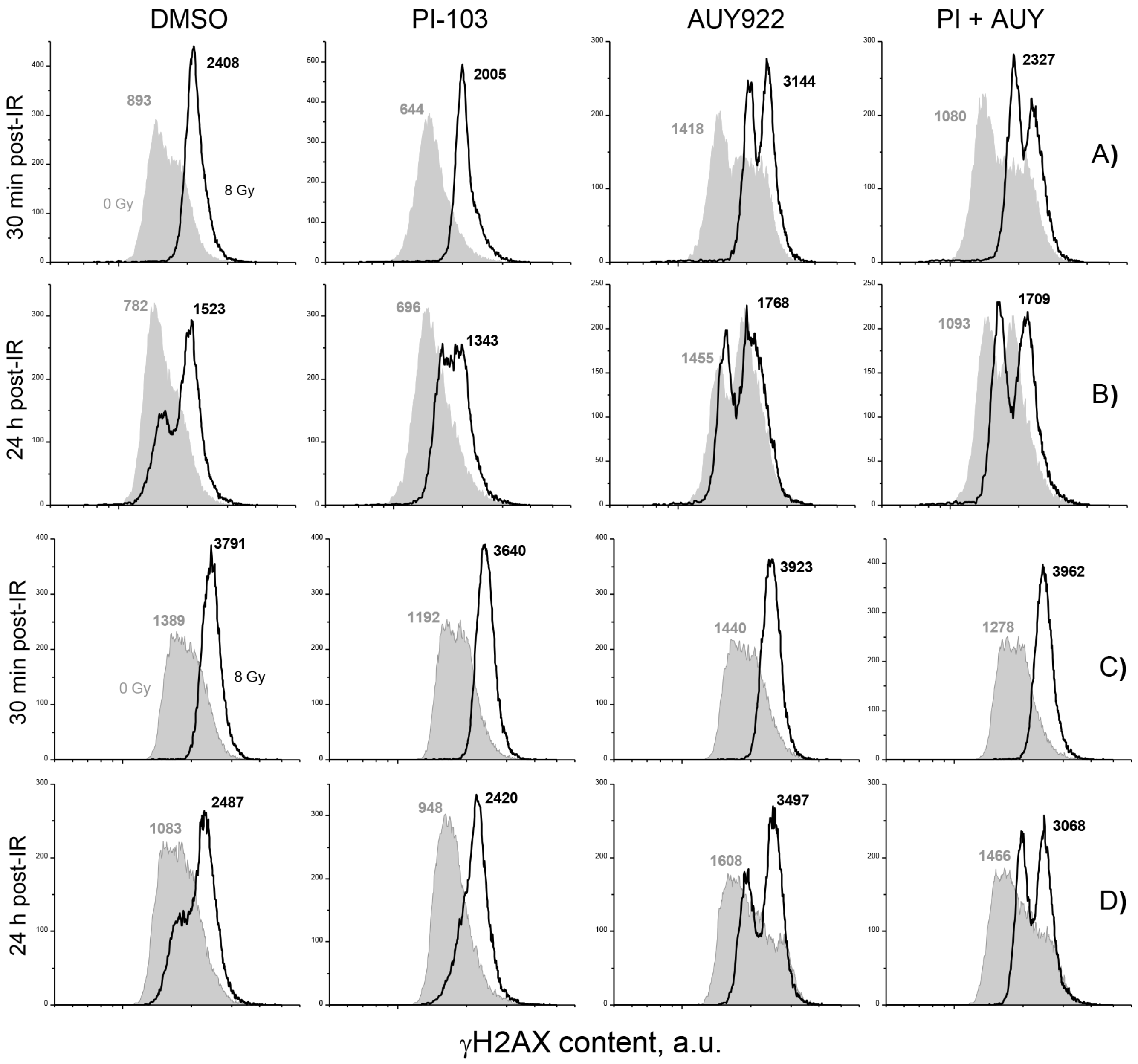

Supplementary Figure S17: Representative flow cytograms of the nuclear histone γH2AX in the colon carcinoma SW48 cells detected 30 min and 24 h after irradiation with 8 Gy. Cells were pretreated with the drugs either 24 h (**A**, **B**) or 3 h (**C**, **D**) before IR. Black and light grey histograms represent irradiated and non-irradiated cells, respectively. Numbers denote the mean histone γH2AX levels for the respective cell samples. The experiment was repeated at least three times.

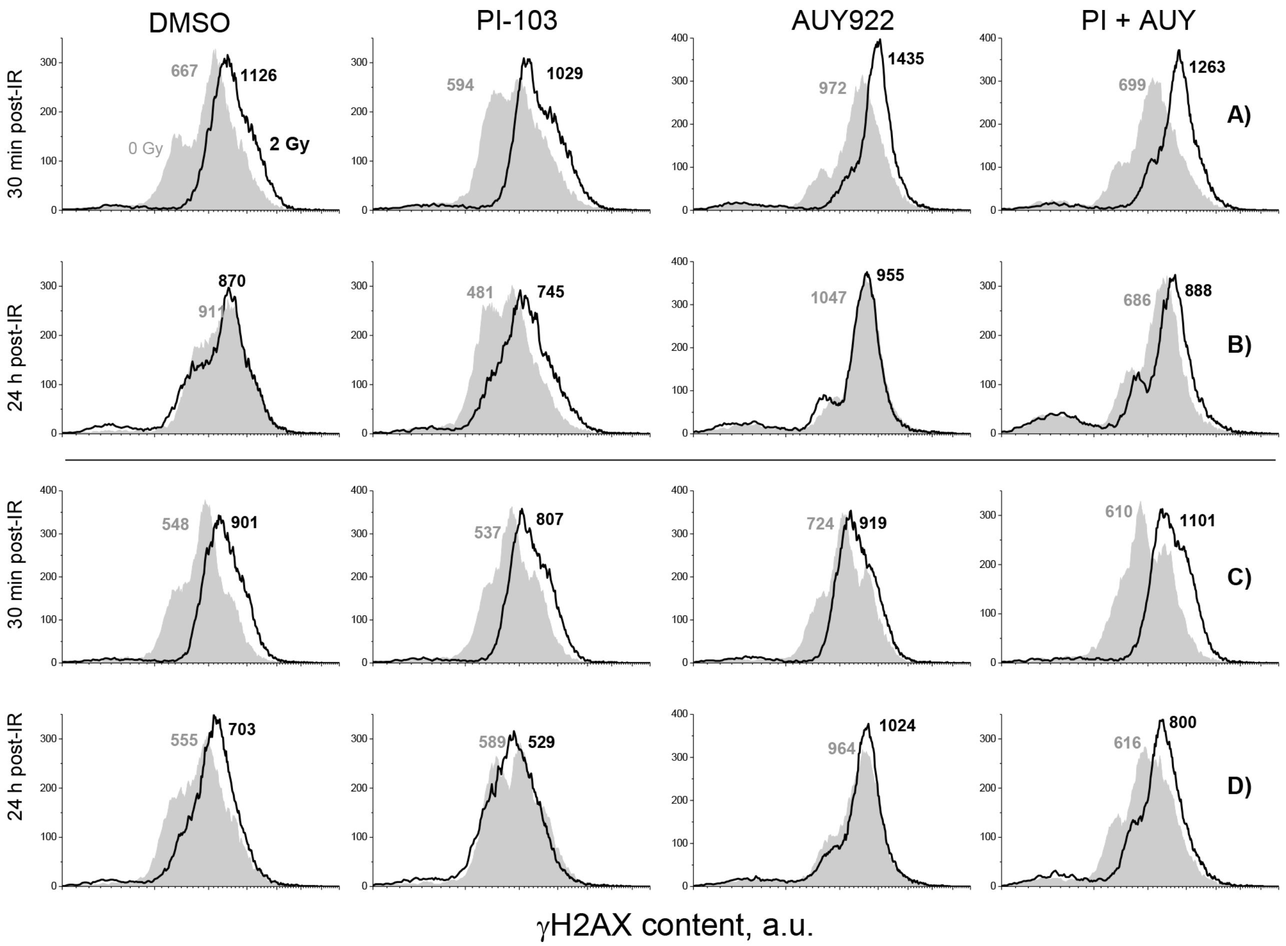

Supplementary Figure S18: Representative flow cytograms of the nuclear histone γH2AX in the colon carcinoma SW480 cells detected 30 min and 24 h after irradiation with 2 Gy. Cells were pretreated with the drugs either 24 h (**A**, **B**) or 3 h (**C**, **D**) before IR. Black and light grey histograms represent irradiated and non-irradiated cells, respectively. Numbers denote the mean histone γH2AX levels for the respective cell samples. The experiment was repeated at least three times.

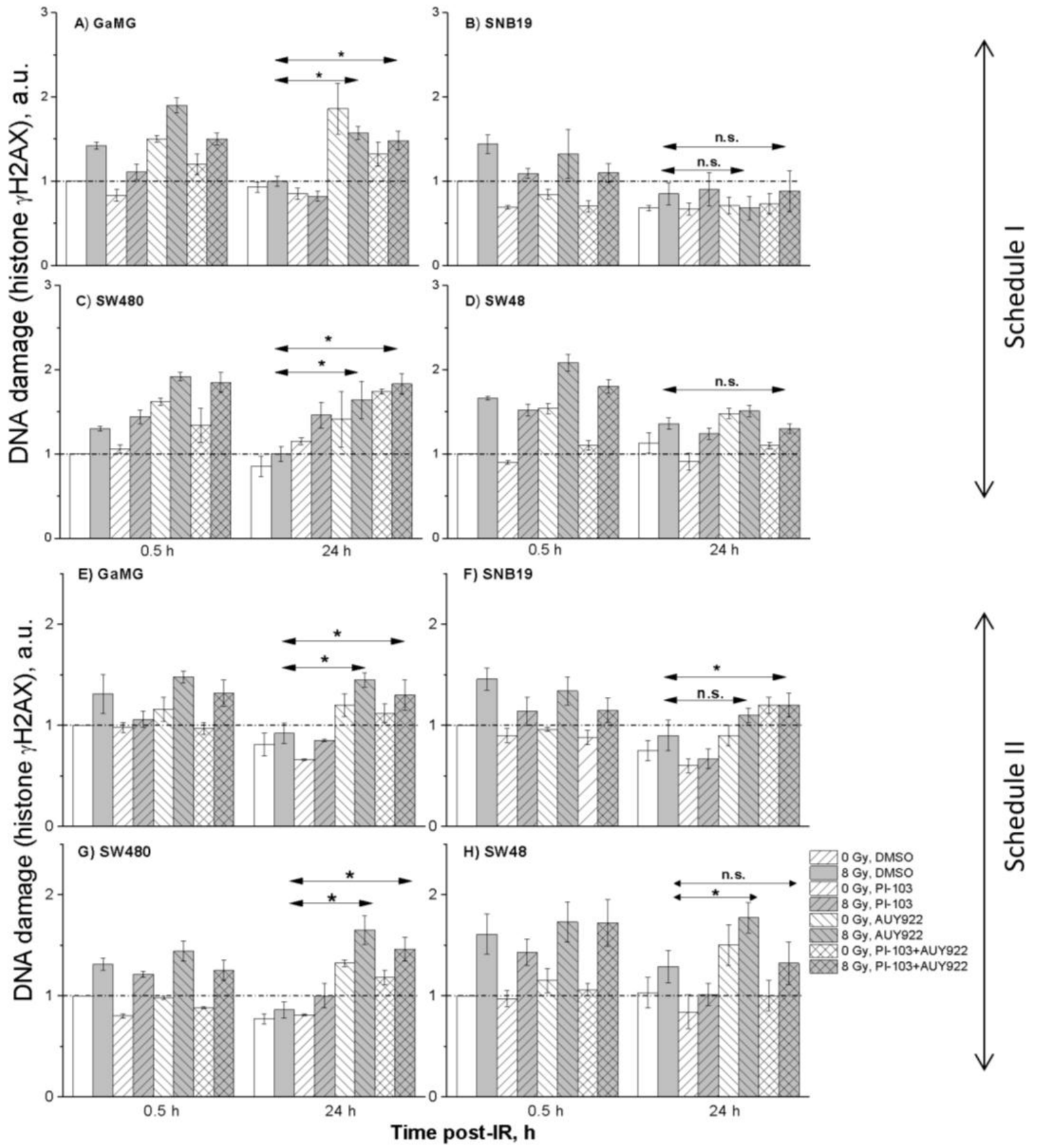

Supplementary Figure S19: DNA damage in non-irradiated and irradiated (2 Gy) GaMG (A, E), SNB19 (B, F), SW480 (C, G) and SW48 (D, H) cells assessed by histone γH2AX and quantified by flow cytometry. Top and bottom halves of the graph refers to the Schedule I and II, respectively. The bar graphs are the means (± SD) of at least 3 independent experiments such as shown in Supplementary Figure S18. The data of each cell line are normalized to the initial γH2AX content (at 0.5 h post-IR) detected in drug-free non-irradiated controls. "a.u." means arbitrary units.

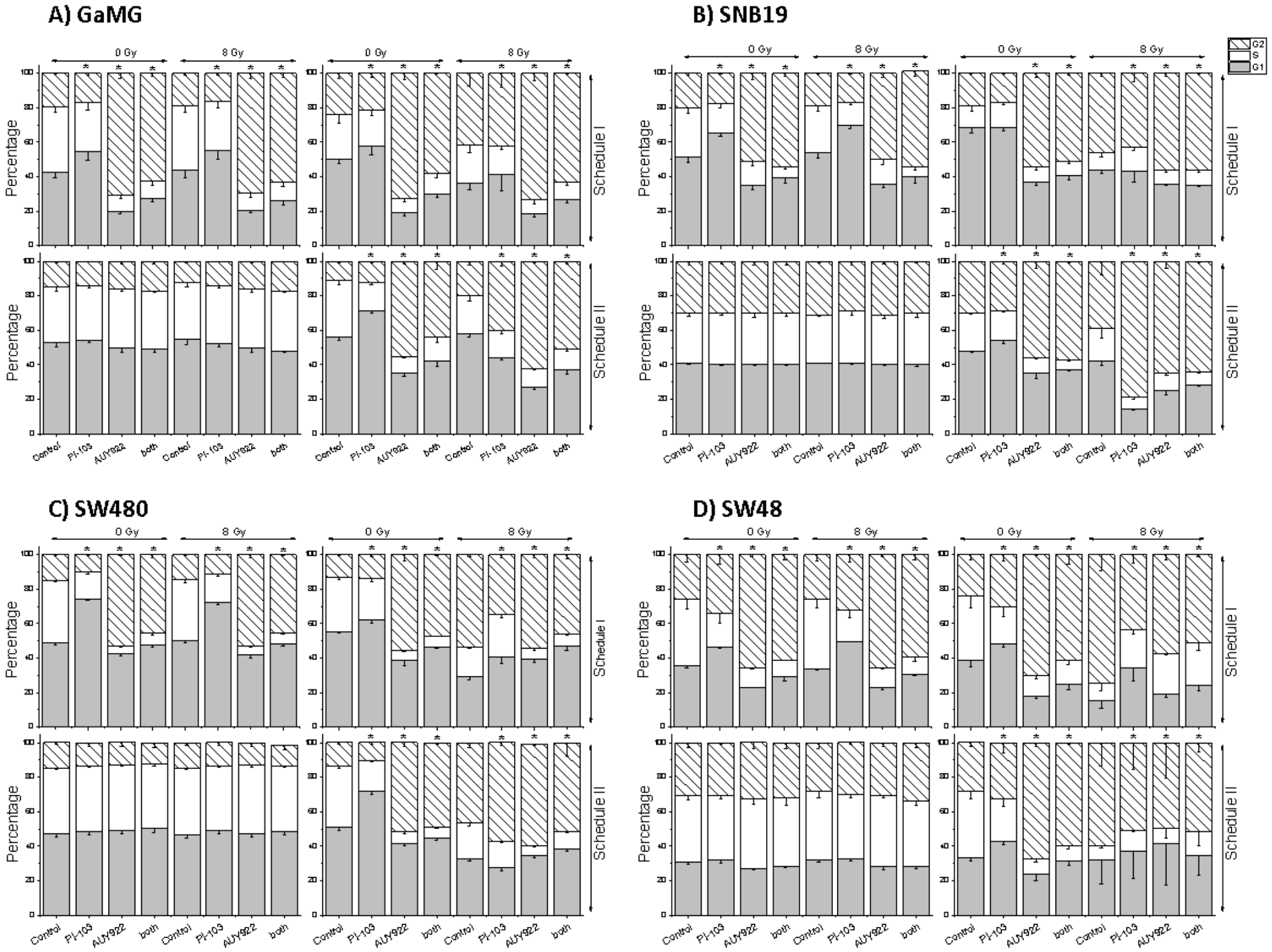

Supplementary Figure S20: Cell cycle-phase distribution in GaMG (A), SNB19 (B), SW480 (C) and SW48 (D) tumor cells treated with PI-103 and NVP AUY922 and IR according to Schedule I and Schedule II. Thirty minutes and 24 h after IR cells were fixed, permeabilized, stained with propidium iodide, and analyzed for DNA content by flow cytometry. Data are presented as means (± SD) from at least three independent experiments. Asterisks show the cell cycle distributions which differed significantly from controls. For detailed description, see Legend to Figure 8.

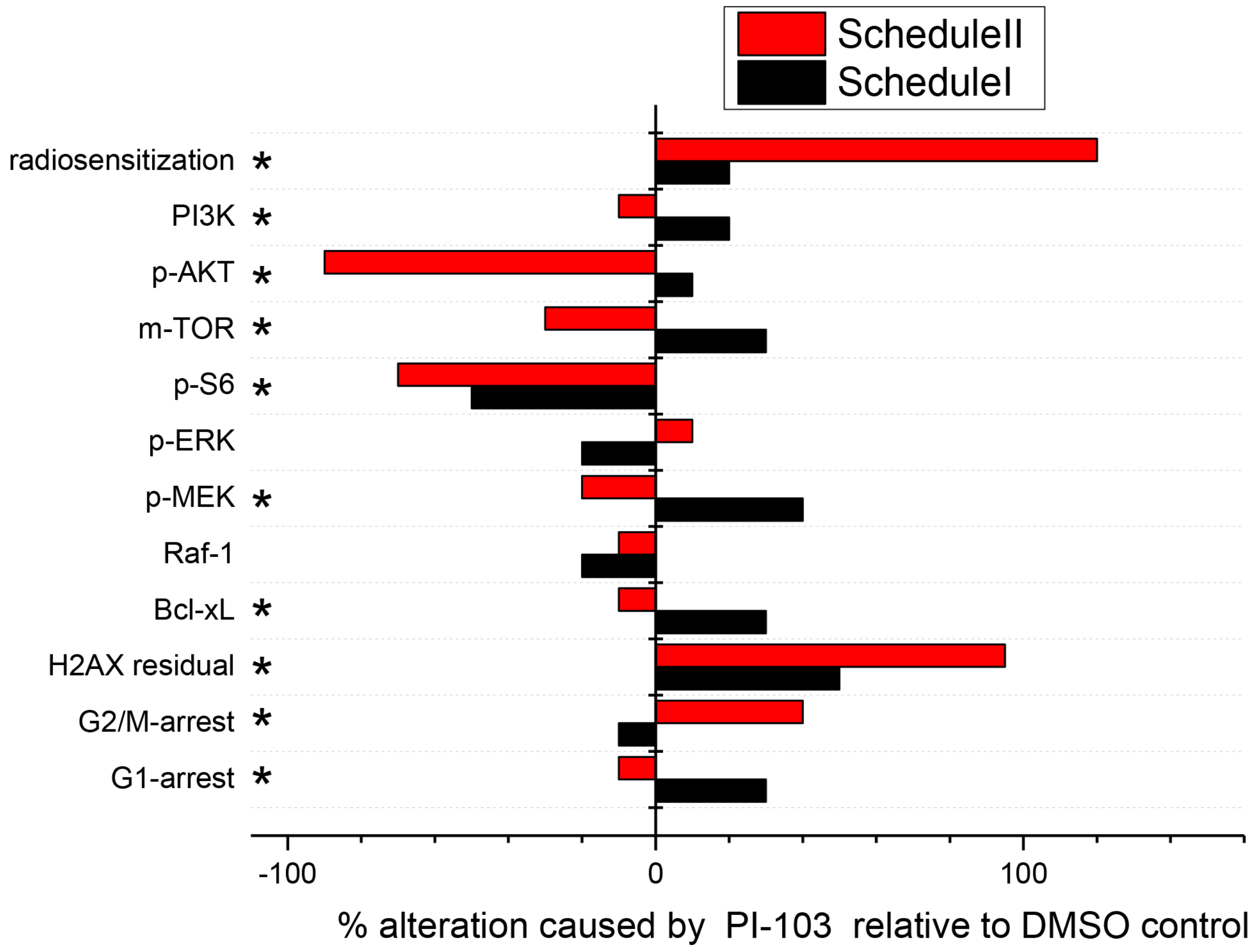

Supplementary Figure S21: Effects of combined PI-103 and IR (8 Gy) treatment on the radiosensitization, marker protein expression, DNA damage and cell cycle arrest induced in tumor cell lines under Schedule I and Schedule II (black and red bars, respectively) detected either 30 min or 24 h (DNA damage, and G2/M arrest) post-IR. The relative changes are given in percent with respect to the corresponding irradiated DMSO controls. The data bars were calculated using the data presented in Figures 1–8, Supplementary Figures S3–S8, S17, S20, and Tables S1, S2. Asterisks show the parameters which differed significantly between the treatments according to Schedule I and Schedule II.

Supplementary Table S1: Cloning efficiencies and radiosensitivity parameters[a] of *in vitro* irradiated tumor cell lines untreated and pretreated with the NVP-AUY922 and PI-103 for 24 h before IR (Schedule I)

Cell line	Plating efficiency	SF2		D_{10} (Gy)[b]		IF_{10}[c] (D_{10} control)/ (D_{10} + inh.)
**GaMG** – contr.	0.24 ± 0.02	0.67 ± 0.05		7.5 ± 0.7		1.0
+ PI-103	0.24 ± 0.03	0.79 ± 0.02		7.7 ± 0.1		1.0 ± 0.08
+ AUY922	0.10 ± 0.02	0.48 ± 0.06	**	4.7 ± 0.2	**	1.6 ± 0.20
+both	0.18 ± 0.04	0.70 ± 0.07	**	5.6 ± 0.3	**	1.3 ± 0.05
**SNB19** – contr.	0.60 ± 0.1	0.52 ± 0.02		4.7 ± 0.3		1.0
+ PI-103	0.50 ± 0.03	0.51 ± 0.05		4.8 ± 0.2		1.0 ± 0.02
+ AUY922	0.36 ± 0.05	0.33 ± 0.01	**	3.5 ± 0.1	**	1.4 ± 0.1
+ both	0.35 ± 0.14	0.47 ± 0.07	n.s.	4.1 ± 0.3	**	1.2 ± 0.2
**SW480** – contr.	0.60 ± 0.1	0.63 ± 0.06		6.4 ± 0.05		1.0
+ PI-103	0.65 ± 0.04	0.61 ± 0.01		5.8 ± 0.01		1.1 ± 0.01
+ AUY922	0.46 ± 0.01	0.50 ± 0.02	**	4.6 ± 0.1	**	1.4 ± 0.02
+ both	0.52 ± 0.02	0.48 ± 0.02	**	4.6 ± 0.1	**	1.4 ± 0.02
**SW48** – contr.	0.50 ± 0.02	0.68 ± 0.03		5.3 ± 0.1		1.0
+ PI-103	0.68 ± 0.07	0.63 ± 0.03		5.2 ± 0.3		1.03 ± 0.1
+ AUY922	0.24 ± 0.02	0.24 ± 0.01	**	2.9 ± 0.1	**	1.8 ± 0.04
+ both	0.46 ± 0.08	0.36 ± 0.01	**	3.6 ± 0.1	**	1.5 ± 0.05

[a]Mean (± SE) from at least three independent experiments;
[b]D_{10} is the radiation dose required to reduce clonogenic survival by 10%;
[c]The growth inhibition factor IF_{10} was calculated as (D_{10} control)/(D_{10}+inh.);
Student's *t*-test was conducted and considered significant at $p < 0.05$ (*) and $p < 0.01$ (**) compared to control survival curve;
n.s. – not significant.
For detailed description, *see* Legend to Figure 1B.

Supplementary Table S2: Cloning efficiencies and radiosensitivity parametersa of in vitro irradiated tumor cell lines untreated and pretreated with the NVP-AUY922 and PI-103 for 3 h before IR (Schedule II)

Cell line	Plating efficiency	SF2		D_{10} (Gy)[b]		IF_{10}[c] (D_{10} control)/ (D_{10} + inh.)
**GaMG** – contr.	0.4 ± 0.1	0.86 ± 0.06		9.7 ± 1.0		1.0
+ PI-103	0.25 ± 0.05	0.76 ± 0.09	n.s.	7.9 ± 0.6	*	1.2 ± 0.2
+ AUY922	0.07 ± 0.01	0.51 ± 0.06	**	4.9 ± 0.2	**	2.0 ± 0.3
+ both	0.09 ± 0.02	0.57 ± 0.07	**#	4.5 ± 0.1	**##	2.2 ± 0.2
**SNB19** – contr.	0.4 ± 0.1	0.64 ± 0.03		7.0 ± 0.3		1.0
+ PI-103	0.4 ± 0.1	0.57 ± 0.05	n.s.	5.6 ± 0.3	**	1.3 ± 0.1
+ AUY922	0.1 ± 0.03	0.50 ± 0.1	**	4.8 ± 0.5	**	1.5 ± 0.2
+ both	0.2 ± 0.1	0.27 ± 0.04	**##	3.2 ± 0.2	**##	2.2 ± 0.2
**SW480** – contr.	0.55 ± 0.2	0.58 ± 0.04		6.3 ± 0.2		1.0
+ PI-103	0.50 ± 0.03	0.46 ± 0.07	*	5.2 ± 0.5	*	1.3 ± 0.1
+ AUY922	0.31 ± 0.04	0.43 ± 0.06	**	4.4 ± 0.4	**	1.5 ± 0.1
+ both	0.37 ± 0.03	0.31 ± 0.1	**#	3.4 ± 0.5	**##	2.0 ± 0.2
**SW48** – contr.	0.44 ± 0.05	0.42 ± 0.05		4.0 ± 0.2		1.0
+ PI-103	0.42 ± 0.04	0.32 ± 0.03	*	3.5 ± 0.2	*	1.2 ± 0.1
+ AUY922	0.18 ± 0.03	0.18 ± 0.03	**	2.6 ± 0.2	**	1.6 ± 0.2
+ both	0.24 ± 0.04	0.18 ± 0.02	**	2.6 ± 0.4	**#	1.6 ± 0.1

For details, *see* Table S1;
Student's *t*-test was conducted and considered significant at $p < 0.05$ (*, #) and $p < 0.01$ (**, ##), where the symbols * and # represent significant difference between survival curves when compared either to vehicle or NVP-AUY922, respectively;
n.s. - not significant.
For detailed description, *see* Legend to Figure 2B.
